# Future-proofing the Cenozoic macroperforate planktonic foraminifera phylogeny of Aze & others (2011)

**DOI:** 10.1371/journal.pone.0204625

**Published:** 2018-10-31

**Authors:** Barry G. Fordham, Tracy Aze, Christian Haller, Abdullah Khan Zehady, Paul N. Pearson, James G. Ogg, Bridget S. Wade

**Affiliations:** 1 Research School of Earth Sciences, Australian National University, Canberra, ACT, Australia; 2 School of Earth and Environment, University of Leeds, Leeds, United Kingdom; 3 College of Marine Science, University of South Florida, St. Petersburg, Florida, United States of America; 4 Department of Earth, Atmospheric, and Planetary Sciences, Purdue University, West Lafayette, Indiana, United States of America; 5 School of Earth and Ocean Sciences, Cardiff University, Cardiff, United Kingdom; 6 Department of Earth Sciences, University College London, London, United Kingdom; Universitat Bremen, GERMANY

## Abstract

The unique macroevolutionary dataset of Aze & others has been transferred onto the TimeScale Creator visualisation platform while, as much as practicable, preserving the original unrevised content of its morphospecies and lineage evolutionary trees. This is a “Corrected Version” (not a revision), which can serve as an on-going historical case example because it is now updatable with future time scales. Both macroevolutionary and biostratigraphic communities are now equipped with an enduring phylogenetic database of Cenozoic macroperforate planktonic foraminiferal morphospecies and lineages for which both graphics and content can be visualised together. Key to maintaining the currency of the trees has been specification of time scales for sources of stratigraphic ranges; these scales then locate the range dates within the calibration series. Some ranges or their sources have undergone mostly minor corrections or amendments. Links between lineage and morphospecies trees have been introduced to improve consistency and transparency in timing within the trees. Also, Aze & others’ dual employment of morphospecies and lineage concepts is further elaborated here, given misunderstandings that have ensued. Features displayed on the trees include options for line styles for additional categories for range extensions or degrees of support for ancestor–descendant proposals; these have been applied to a small number of instances as an encouragement to capture more nuanced data in the future. In addition to labeling of eco- and morpho-groups on both trees, genus labels can be attached to the morphospecies tree to warn of polyphyletic morphogenera, and the lineage codes have been decoded to ease their recognition. However, it is the mouse-over pop-ups that provide the greatest opportunity to embed supporting information in the trees. They include details for stratigraphic ranges and their recalibration steps, positions relative to the standard planktonic-foraminiferal zonation, and applications as datums, as well as mutual listings between morphospecies and lineages which ease the tracing of their interrelated contents. The elaboration of the original dataset has been captured in a relational database, which can be considered a resource in itself, and, through queries and programming, serves to generate the TimeScale Creator datapacks.

## Introduction

Foraminifera comprise a very useful component of living and fossil zooplankton [1], providing, for example, primary indices for global chronostratigraphy particularly from the Cretaceous to Cenozoic [2], and key proxies for paleoclimatological parameters [3]. The rich fossil record of planktonic foraminifera also makes them superb case examples for the study of evolution through deep time [4]. This is especially so for the Cenozoic, as their present diversity of approximately fifty species grew from just a few that survived the end-Cretaceous major extinction event ([5], though see, e.g., [6]).

Aze & others [7] (below, abbreviated as the “2011” study, dataset, trees, etc.) provided a critical compilation of ancestor–descendant relationships and time ranges among most living and fossil Cenozoic planktonic foraminifera, those belonging to the monophyletic macroperforate group. Very importantly especially for macroevolutionary studies, that work reconfigured the established morphospecies taxa to recognise paleobiological lineages, thus attempting to eliminate taxonomic effects arising from “pseudoextinction” and “pseudospeciation”. Their linked and stratigraphically dated phylogenies of morphospecies and lineages for Cenozoic macroperforate planktonic foraminifera, the first attempt at both concepts published in decades, now provides a valuable dataset to more effectively explore a broad suite of topics, be it say, macroevolutionary dynamics, reliability of chronostratigraphic indices, or phylogenetic interpretation of the genome.

The importance of the 2011 dataset would seem incontestable, but continuing to make use of it into the future presents some practical challenges. The chief hurdle is that ages used in the paper, including stratigraphic ranges and divergence levels of taxa, were presented as dates (in Ma, i.e., 10^6 years ago; abbreviations Ma and Myr follow [8]) compiled from a large number of sources and converted to the time scale of Cande & Kent [9] as used by Berggren & others [10]. As standard international geological time scales are progressively updated, future retention of these ages (that is, as stratigraphic levels, as opposed to Ma numbers) will depend on their relationships to the time scales employed by each of these sources. So, in order to maintain currency of Aze & others’ dataset—minimally, to avoid obsolescence of its ages—we now need to augment it with time-scale details specific to each taxon, prior to progressively recalibrating the dates through the relevant time-scale schemes. As onerous and mundane as this task is, it is made more challenging by the special case of planktonic foraminifera in that they provide the finely demarcated datums for standard Cenozoic zonations. One aspect of the extra care needed because of this, is that the Aze & others compilation favoured phylogenetic sources and, for some datum taxa, the ages from these sources do not match those adopted for the zonations. A second aspect is that the ancestor–descendant relationships for morphospecies compiled by Aze & others relied upon a homotaxy (consistent stratigraphic order [11], e.g., [12]) supported by the dates employed, and these relativities will also need to be maintained through the changes in time scales. Similarly, we will need to ensure that the links between ages for the morphospecies and the lineages proposed in the paper are kept intact.

In this study we have chosen to maintain the currency of Aze & others’ trees by reconstructing them for the evolutionary-tree function of the visualisation suite, TimeScale Creator (Table 1 row 1). In so doing, greater transparency has been obtained for the ages, including explicit indication of time scales applied, detailing of recalibrations along the time-scale series leading to GTS2016 (Table 1 row 2), and, for those that serve as datums, comparisons with dates used by both the low-latitude Cenozoic planktonic-foraminiferal zonation of Wade & others [13] and by the Geologic Time Scale releases starting with GTS2004. Other information tabulated in Aze & others’ paper has also been incorporated, including morphological, ecological and geographical summaries, and stratigraphic ranges from the Neptune deep-sea microfossil occurrence database [14–16]. Also, while presenting Aze & others’ study, we have detected a need to further elaborate their dual employment of morphospecies and lineage concepts, given some misunderstandings of their macroevolutionary significance that have ensued.

**Table 1:** Extended remarks^a^.

1. TimeScale Creator (TSCreator, TSC) [17, 18] is an initiative of the Geologic TimeScale Foundation Inc. (West Lafayette, Indiana), based at the Department of Earth, Atmospheric, and Planetary Sciences, Purdue University. Major upgrades to TSCreator are released concurrently with each major release of the International Commission on Stratigraphy’s Geological Time Scale (latest is [19]). The current TSCreator Version 7.3 [20] corresponds to GTS2012 [21] and the selective updates constituting GTS2016 [22]. Updates and calibrations of columns are incorporated as appropriate, typically several times a year.
2. The relevant time scale series is discussed in § From the tables: derived data and information, and datapacks. The scales (as acronyms) referred to herein based on planktonic-foraminiferal zonations are: BKSA95 [10], BP05 [23], and WPBP11 [13] (calibrated to CK95 [9] or GTS04 [24]). WPBP11 (GTS04) links to the GTS series consisting of GTS2004 [24] and GTS2012 [21], with selective updates via the intermediary GTS2008 [25] and GTS2016 [22]; GTS2008 is not needed herein as its relevant chronology did not differ from GTS2004. The "GTS" label was appropriated from the original GTS1982 [26] and GTS1989 [27].
3. “Corrected”, “updated”, and “revised”. An explanation is needed for the words we use to enunciate changes made to the 2011 trees, both for the transfer to the Corrected Version as well as for future changes that are anticipated. (a) Select changes within the trees. Firstly, there are some differences between the 2011 trees and the TimeScale Creator trees that are not changes but enhancements. These include, for example, a few instances where the transferred trees discriminate parts of stratigraphic ranges that the nominated sources considered uncertain. Then there are clear-cut corrections to the data of the 2011 trees, for instance, dates mistranscribed (though only slightly different) from the sources, and misquoted sources for ages (often a result of the 2011 paper tabulating only a single source entry for both start and end dates). The Corrected trees also include changes better labeled amendments which were, for example, needed to obtain consistency with the dates employed by Wade & others’ [13] zonation, which was erected in parallel with and partially independently from the Aze & others study. The variety of contexts in which changes have been made for the transfer from the 2011 trees precludes a worthwhile terminology for the changes, but the important point to note is that all corrections, amendments, and enhancements are consistent with the intensions and contemporaneity of the 2011 study. And the back-end database preserves all original data, employing additional columns to house all changes, including comments on them, ensuring transparency and enabling tracking of changes. (b) Recalibrations. A key outcome from the TimeScale Creator transfer has been to position the 2011 trees for past and future updates to the international geological time scale. This positioning has required enhanced database design and more data, as well as corrections and amendments: a variety of select changes within the trees. The dates within the trees are then set against the employed time scales, and all can be recalibrated to a common base, the most recent scale in 2011 being GTS2004. This stage of the Corrected version can be considered as that which is intended for preservation of the 2011 trees as an historical case example. The only future changes envisaged for the preserved 2011 trees are not corrections or amendments but updates to later time scales. Herein the updates to GTS2012 and GTS2016 have been invoked, and it is anticipated that the next would be to GTS2020. These recalibrations are not select changes but changes applied throughout each tree, and each update preserves the internal consistency within the 2011 case example while maintaining its currency. (c) Revisions. Like any active research, the 2011 trees were outdated as soon as they were finalised—especially as they were “firsts” in many respects—and will need to be revised to incorporate later and, possibly, missed or misunderstood research. Obviously this will include potentially major changes to taxonomy, time ranges, and ancestries. The most obvious incentive will come from remaining outputs of the Paleogene Planktonic Foraminifera Working Group and commensurate activities underway for the Neogene. Below, § Following on alludes to this aspect. So the Corrected Version presented here incorporates corrections, amendments, and enhancements, and positions it for time-scale updates, while maintaining the integrity of the 2011 case example. Of course, “Corrected” does not adequately imply all this, but its main purpose it to remind us that it does not constitute a revision.
4. Stainforth & others’ [28] survey of Cenozoic planktonic-foraminiferal index forms employed less than half of the 339 species-group taxa of the 2011 study. Plankton Stratigraphy’s [2] chapters on low-latitude Cenozoic planktonic foraminifera [29, 30] amounted to ~242 macroperforate species-group taxa (318 taxa from the 2011 study would have been available at the time of the Plankton Stratigraphy surveys), the smaller number presumably a result of a somewhat broader approach to taxa as well as a focus on those considered more-stratigraphically useful. Kennett & Srinivasan’s [31] Neogene Atlas employed a similar number of species-group taxa to that of the 2011 study but its coverage was restricted to the Neogene. Fordham’s [32, 33] study of Cenozoic planktonic foraminifera provides an opposite comparison in regard to numbers of species-group taxa employed. He listed ~900 available species-group names as potential macroperforate phena (taking into account 31 Neogene names indicated as not worth distinguishing i.e. considered synonymous phena). Given that Fordham’s compilation of Paleogene names was somewhat uncritical (and so could have included significant numbers of synonymous or unavailable phena), ~750 may be a more realistic estimate for his study (this would reflect Fordham’s approach to encourage the finest practicable discrimination for phena in order to maximize their potential for biostratigraphic or paleoecological/biogeographic application). This number is of course still over twice the 308 morphospecies from the 2011 study available at that time to Fordham and reflects the major difference between Fordham’s phena and the morphospecies of the Working Group and the 2011 study.
5. Estimates of the number of Blow’s [34] macroperforates were made manually herein. Of his Checklists: all macroperforates were counted from the Late Middle Eocene—Recent Checklist 1–228; for the Danian—Oligocene Checklists 229–687 and 688–760 (informa), macroperforates which were "Plotted on the Range Charts" (indicated by †) were counted, excluding taxa duplicating the 1–228 Checklist. The resulting totals were 398 macroperforate species-group taxa, reduced to 364 species and subspecies when informa were excluded, within which 270 were considered species. These manual counts are not readily comparable, though may very well be approximately compatible, with the 262 morphospecies of Paleogene planktonic foraminifera (macroperforate and nonmacroperforate) of Blow reported by Pearson (page 117 in [35]).
6. As already noted, Blow did recognise ~270 nominal species, a similar total to the 281 morphospecies available to him that were recognised in the 2011 study, but these similar totals are largely coincidental as the Working Group and the 2011 study included as their morphospecies a large number of Blow’s subspecies raised to species.
7. This nomenclatural approach may have been part of a trend away from more complicated nomenclatures (including multiple infraspecific categories, subgenera, etc.) and toward greater reliance on simply employing the most trivial epithet [as highlighted by [34], see page 753]. This may have been encouraged by a number of factors, including: the increasing complexity of phylogenetic proposals and the concomitant graphical incentive to express taxa simply as specific or subspecific epithets (from e.g., [36]), including graphical devices such as Pearson’s [37] plexigrams; the increasing use of lineages to define genera or subgenera (from e.g., [38–41]), lessening the need to express this phylogenetic information infraspecifically; and analogously the growing appreciation of the paleoceanographic complexity of plankton water masses (e.g., Text-fig 2 in [42]) which probably tended to undermine the perceived validity of complicated infraspecific taxonomies.
8. Cladistic case studies on planktonic foraminifera include: [43–47]. Further discussions regarding limitations of applying cladistics to planktonic foraminifera include: [37, 48–50], Pearson in [51], page 907 in [7].
9. [52], for example, reviewed most molecular studies of planktonic forms then available. [53] is an example of a phylogenetic study based on stratophenetics but integrating molecular results.
10. The 2011 study’s usage of morphospecies (following Pearson, see § Morphospecies tree) is not captured well by Mayden’s [54] listing. Comparison with Mayden’s Morphological category is undone by the dimensionality conferred by its lineage context; it is closest to his Successional category but, contrary to Palaeospecies and Chronospecies, it is delineated morphologically (“vertically”, not “horizontally”, against a stratigraphic or time abscissa).

^a^ See references in the text to these entries

All of the information has been organised into a back-end relational database, which becomes a valuable resource in itself. Much of this information is displayed in pop-ups associated with each taxon on the TimeScale Creator tree, the contents of which are entirely generated from the database.

For this Corrected TimeScale Creator Version of the 2011 trees, the intention is to preserve the content of the trees in its original unrevised form: the original selection of ages and relationships in the trees has been faithfully transferred, with corrections only where needed. This is meant to provide a TimeScale Creator tree that is consistent with follow-on studies which have already applied data from the original paper, and which will establish an historically representative depiction of the original trees that can then serve as a base for comparison with future updated TimeScale Creator versions (for this context, usage in this paper of terms like “corrected”, “updated”, and “revised” is explained in Table 1 row 3).

The back-end database also positions the 2011 trees well for adjustments, supporting not just time-scale updates but future revisions to the phylogenies and stratigraphic information. This is particularly pertinent for organisms such as planktonic foraminifera which, despite their rich fossil record, are especially vulnerable to phylogenetic reinterpretation as their limited morphologic palate makes them highly susceptible to homeomorphy, iterative evolution, cryptic speciation, and the like [4, 52]. It is anticipated that a major revision of the trees will incorporate information from the recently published “Atlas of Oligocene planktonic foraminifera” [55].

## The trees of Aze & others (2011)

**Fig 1:**
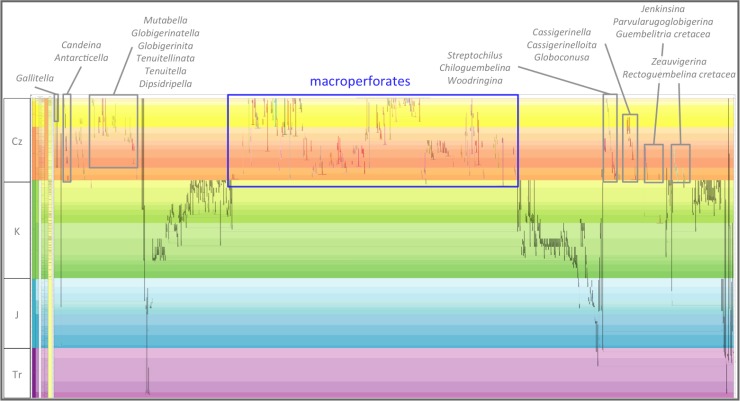
Cenozoic macroperforate planktonic foraminifera in relation to other Cenozoic planktonic-foraminiferal groups. Overall view merely to convey major ancestor–descendant groupings, against a mock-up phylogeny of all planktonic and related benthic foraminifera from their Mesozoic origins. Drawn using the evolutionary trees function of TimeScale Creator from a database compiling ancestor–descendant proposals from various sources. The genus and species labels, for Cenozoic planktonic-foraminiferal groups other than macroperforates, are merely indicative (note that the Oligocene Atlas [55] updates these micro- and medio-perforate taxa and their classification).

The subject of the 2011 study were all of the Cenozoic species of the macroperforate planktonic foraminifera (the genera *Hastigerina* and *Orcadia*, with their distinctive triradiate barbed spines, were excluded from the study as stratophenetic evidence for their origins was considered too poor). The macroperforates constitute some 80% of the 45 or more morphologically distinct living species of planktonic foraminifera (molecular studies point to a diversity [52] within living planktonic foraminifera [1] which is proving challenging to evaluate and document [56]). They make up a similar portion of Cenozoic planktonic foraminifera (Fig 1). Macroperforates are typified by calcareous tests with at least inner wall surfaces punctuated by distinct pores greater than 1 μm in diameter [57–59] (as employed in [7], macroperforate = medioperforate + macroperforate of [58], and normal perforate of [59]). At least as a Cenozoic set, they are considered a monophyletic group descended from the immediate common ancestor of two sister lineages that survived the end-Cretaceous extinction event.

The 2011 study produced two phylogenetic trees for Cenozoic macroperforates (Figs 3–5 and Appendices 2 and 3 in [7]): a morphospecies tree, critically compiled from the literature (basic source data were given in Table S3 of Appendix S1 in [7]); and a lineage tree, drawn indirectly from the morphospecies tree but, rarely outside vertebrate paleontology, interpreting lineages as comparable to biological species extended through geologic time.

### Morphospecies tree

**Fig 2:**
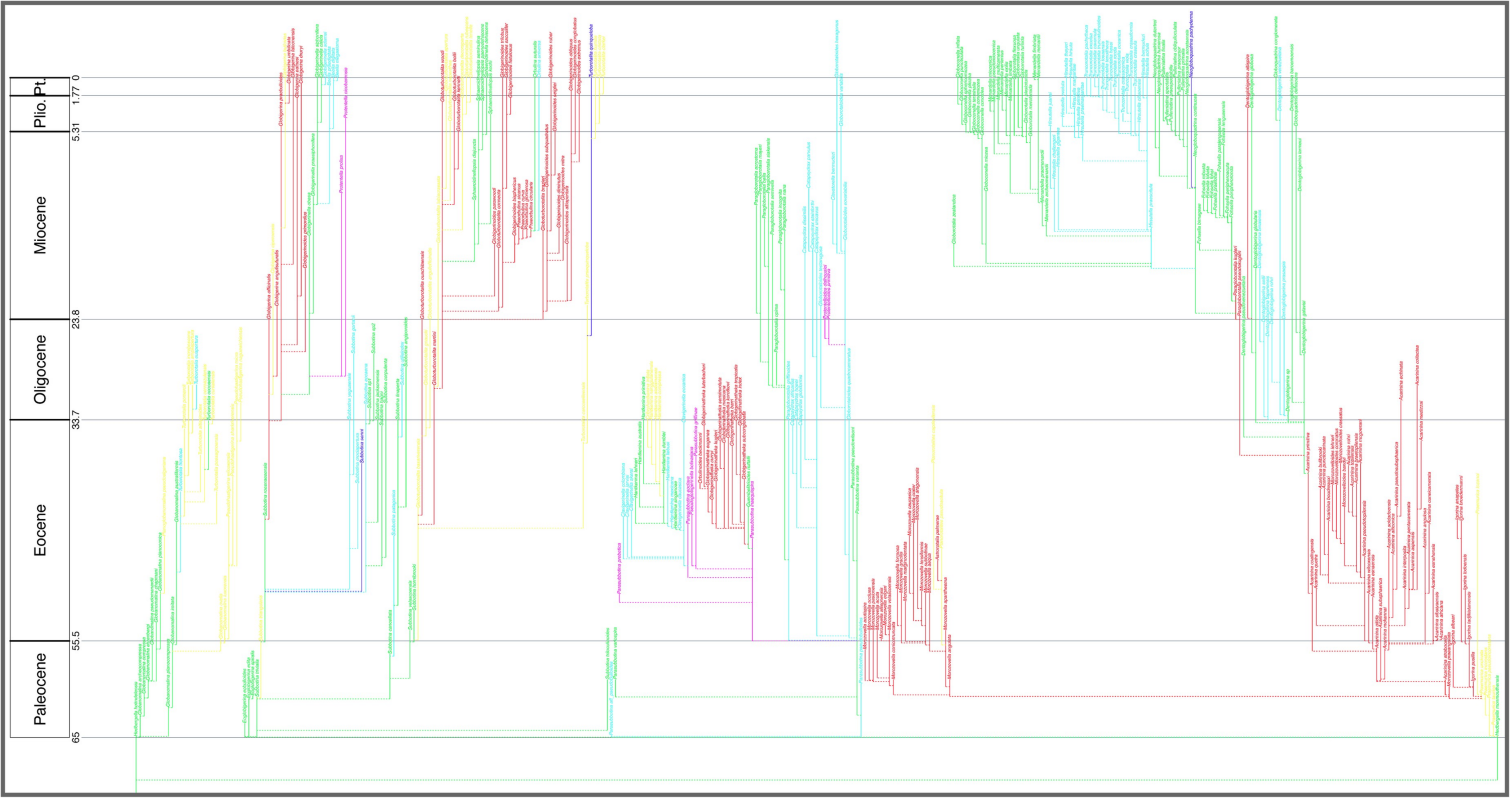
Aze & others’ (2011) tree of morphospecies of the Cenozoic macroperforate planktonic foraminifera. Their Budding/Bifurcating Morphospecies Phylogeny (Appendix S2 in [7]): overall view merely to convey the scale and temporal extent of ancestor–descendant groupings; ranges and morphospecies labels coloured by ecogroup (see original for details, which can be zoomed in to read labels and ranges, especially those of yellow Ecogroup 2 which are indistinct here).

For the morphospecies tree (Fig 2) the Paleocene and Eocene parts were compiled from the Atlases of the Paleogene Planktonic Foraminifera Working Group of the International Subcommission on Paleogene Stratigraphy [5, 60]. Several sources were employed for the post-Eocene portion, including Stewart’s study for Neogene globorotaliids [46], though the Neogene Atlas of Kennett & Srinivasan [31, 61] was the dominant overall source for Neogene taxonomy, stratigraphic ranges, and ancestor–descendant relationships. Sources specific to each morphospecies (Table S3 of Appendix S1 in [7], especially “Date reference”) also contributed to details of the tree.

The morphospecies of the 2011 study were primarily guided by the morphospecies concept of the Paleogene Working Group. This concept encapsulated more than Cain’s (page 82 in [62]) use of the term for specimens morphologically distinct from closely related forms. They viewed morphospecies as segments of lineages (Fig 6 in [35] and Fig 2 in [37]), so in that sense analogous to (though employing demarcation criteria different from) chronological segments which have been termed paleospecies [62] or chronospecies [63]. This taxonomic segmentation of lineages into morphospecies, for mostly biostratigraphic purposes, was acknowledged to be necessarily artificial. However, it seems that the selection of species taxa within a lineage context allowed the Working Group to rationalize the varied previous approaches to taxonomy of planktonic foraminifera tried over several decades. They operationalized this in two main ways. Nomenclaturally, they employed only binomina (*Genus species*), whereas previous schemes often involved a complex array of species, subspecies, and other infraspecific categories, classified within genera and subgenera. Taxonomically, they recognised (as species) only species-group morphotaxa considered “readily communicable between workers” (page 16 in [60]). These approaches resulted in a significant distillation of previous usage. In particular, the 339 macroperforate morphotaxa recognised in the 2011 study can be contrasted with those of the three-volume tome of Blow [34], the only comparable coverage of Cenozoic planktonic foraminifera (Table 1 row 4). Even though only 281 morphotaxa names of the 2011 study were available to Blow, he employed ~364 macroperforate species and subspecies (Table 1 row 5), totaling ~400 morphotaxa once his “informa” are included (Table 1 row 6). The lesson from this comparison would seem to be that the approach of morphologically segmenting lineages results in somewhat broader species-group taxa which incorporate quite a number of those morphotaxa of Blow, reinterpreted as paleoenvironmental or biostratigraphic variants.

A case can therefore be made that employing morphospecies as morphological segments of lineages, as invoked by the Paleogene Working Group and applied to the 2011 study, can help stabilise taxonomy and provide taxa that can be both evolutionarily meaningful and of practical application for biostratigraphy and paleoecology/biogeography. A lineage divided into overlapping morphologically intergrading morphospecies (Fig 6 in [35] and Fig 2 in [37]) could of course be paleobiologically misleading, but it can be argued that it is a reasonable compromise. And in the case of, for example, a lineage which remains relatively unchanged morphologically through an extended interval of deep time, the lineage will sometimes be represented by a single morphospecies, and so the morphospecies would then approximate a (biological) species (an example is the 9+ Myr of the *Globoturborotalita* lineage in the Ypresian–Lutetian of the Eocene, constituted solely by *G*. *bassriverensis*, prior to the appearance of *Turborotalita carcoselleensis*, *G*. *martini*, and *G*. *ouachitaensis*; see Fig 6.2 in [64]). Within this context, the application of species binomina to all morphospecies, rather than employing multiple species-group categories, attains considerable justification. The Paleogene Working Group (starting with the Paleocene Atlas [5]) appears to have been the first to apply species binomina to all morphotaxa on such a scale, at least to planktonic foraminifera (Table 1 row 7).

### Ancestor–descendant relationships

The 2011 study did not directly present evidence for the proposed ancestor–descendant relationships between morphospecies but relied upon its main sources and, to a lesser extent, those specific to individual morphospecies (see § Morphospecies tree, first paragraph). This literature reflects extensive application of planktonic foraminifera to biostratigraphy by very large numbers of observers in industry and academia since at least the 1950s. The dominant phylogenetic tradition practiced by these communities applies stratophenetic [65] approaches to usually detailed stratigraphic sequences of typically abundant collections of well-preserved tests of planktonic foraminifera, leading to the interpretation of lines of descent between closely spaced successive collections. In this way, phylogeny is typically reconstructed well below the level of species-level taxa, based mainly on direct microscopic observation of very high numbers of stratigraphically organized specimens [66]. Although the ancestor–descendant relationships proposed are usually supported by selective imaging and noting of morphological changes, it is impractical to publish the information amassed. As a result, evidence actually presented is usually indicative at best, and published proposals can appear to be simple unsupported claims, but this would be a misreading of the evidence accumulated (a more systematic/structured approach to routinely documenting such evidence has been trialled by [67]). A small proportion of relationships proposed have been backed by case studies applying detailed and sophisticated morphometric and imaging approaches (see, e.g., Appendix in [68]).

It is important to appreciate that this, what could be termed “record-rich”, approach that is routinely applied in planktonic-foraminiferal studies is necessarily a variant from standard stratophenetics. Collections available for the latter case studies (typically on vertebrates) usually employ samples of tens of specimens in outcrop successions in which the fossil recovery is quite reliable but nonetheless scattered both up and along section. There, the plausibility of lines of descent typically relies upon the interplay between moderately closely spaced stratigraphic sampling and statistically useful collections of biometric measurements of the complex evolutionarily distinctive morphologies provided especially by skulls. Planktonic foraminifera, on the other hand, have tests with a much more limited observable morphological variety, highly susceptible to apparent evolutionary reemergence [69]. Here, the plausibility of lines of descent comes from the continuity and richness of the record provided typically by deep-sea sequences of planktonic foraminiferal oozes, almost continuous up and along section, in which the only practical limit on collection size is in the hands of the sampler.

So record-rich stratophenetics is a relatively low-tech method of phylogeny reconstruction that emerges from relatively routine microscopic observations of specimen-rich stratigraphically closely connected collections. This record-rich aspect brings forth a key methodological feature not usually attributable to “Gingerichian” stratophenetics: the stratophenetic method of phylogeny reconstruction is the only available approach that can adequately exploit the rich stratigraphic record of planktonic foraminifera. As a result, cladistics and molecular methods of phylogeny reconstruction, the other approaches that have become standard for most biological groups in recent times, continue to play only secondary roles to stratophenetics for moderately comprehensive phylogenetic studies of planktonic foraminifera. Despite major breakthroughs in especially molecular methods, these approaches suffer from the necessarily highly restricted sampling of specimens involved. This is of a taxonomic kind for cladistic approaches: studies are restricted to recognised taxa or operational taxonomic units and these represent only a generalised and simplified selection and abstraction from the rich population-level collections available to stratophenetics (Table 1 row 8). For molecular approaches this restriction is temporal, as only representatives from living lineages can be included and, then, only those that can be captured (Table 1 row 9). These ancillary approaches thus serve to inform, rather than displace, stratophenetics. Nonetheless, they can highlight key shortcomings of the reliance of stratophenetics on fossils, particularly the limited discrimination that foraminiferal test morphology provides to address cryptic populations/species/etc. Another shortcoming applies when the record is poor, particularly at the origins of those clades exhibiting fundamental but often-subtle evolutionary change in, for example, test-wall microstructure; here, investigators steeped in stratophenetics can easily overreach when attempting to connect across larger-than-anticipated gaps (stratigraphic or morphologic) in the record.

### Lineage tree

**Fig 3:**
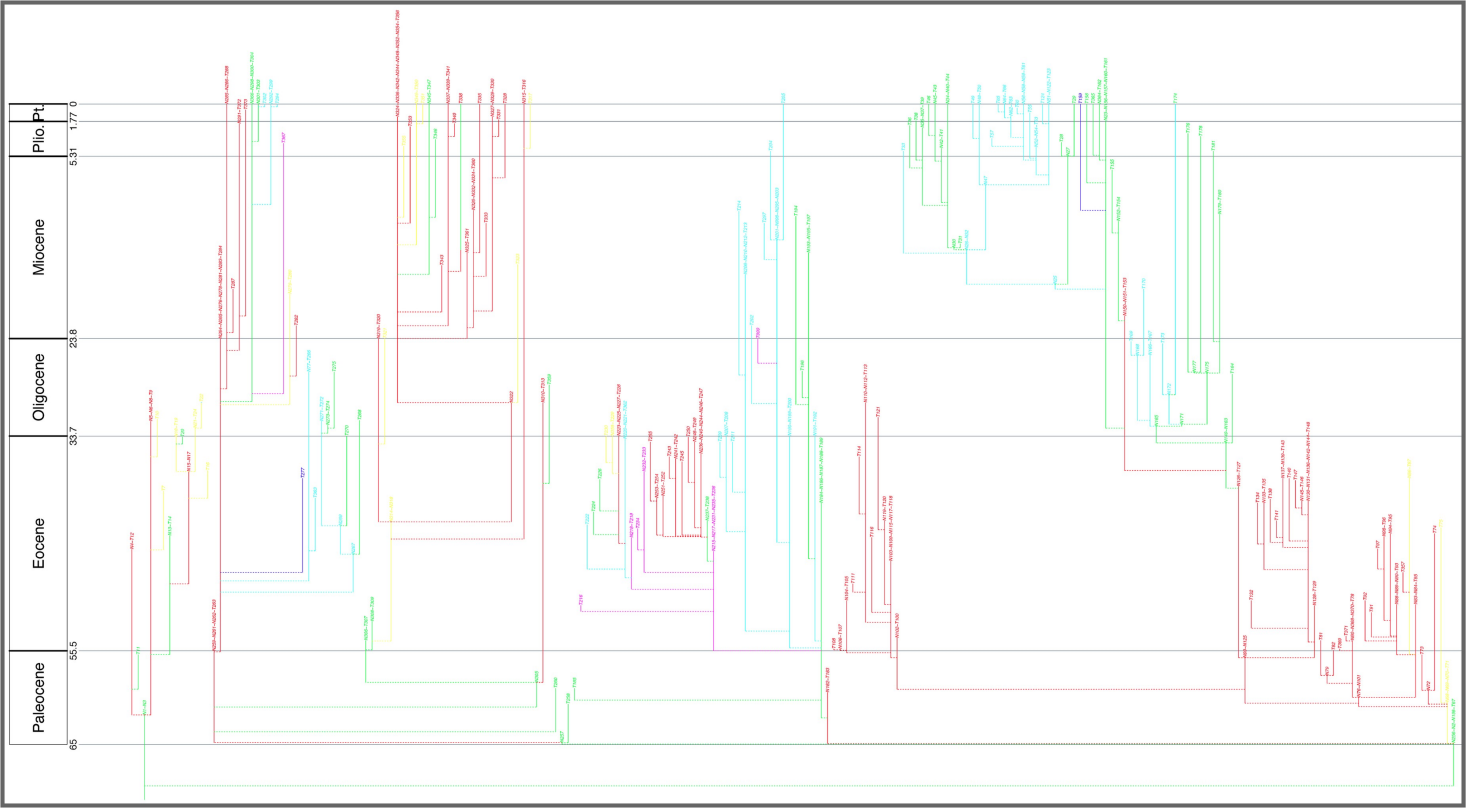
Aze & others’ (2011) tree of lineages of the Cenozoic macroperforate planktonic foraminifera. Their Budding/Bifurcating Lineage Phylogeny (Appendix S2 in [7]); otherwise as for Fig 2.

The lineage tree of the 2011 study was newly proposed (Fig 3). It was derived manually from the morphospecies tree by taking account of intergradations observed between morphospecies (Text-fig 3 in [37] and Fig 2 in [7]) from stratigraphic sections worked on by the authors as well as from reports in the literature, including Stewart’s study of Neogene globorotaliids [46]. The evidence specific to these intergradations was, however, not presented. The resulting 369 lineage segments were not named taxonomically but, rather, labeled with codes (e.g., “N88”, “N89”, “N90”, “T93”), which were concatenated to form labels (e.g., “N88-N89-N90-T93”) for the corresponding 210 whole lineages (see Fig 2 in [7]).

The only precedent for the 2011 study’s lineage tree is the tree of all Cenozoic planktonic foraminifera by Fordham (Text-fig 4 in [32] and Text-fig 2 in [33]), similarly constructed (from his phena: analogous to, but not equivalent to, the morphospecies of the 2011 study; see Table 1 row 4). Fordham presented his evidence, for intergradation within “species clusters” in the sampled sections, as Species–Phenon charts (Tables 3 and 4 in [33]), and also referred in the text to other evidence from the literature. Even taking into account the slightly shorter list of species-group taxa available to Fordham at that time, his was a much more simplified tree containing only 110 macroperforate lineage segments (each labeled a separate species). The main reason for this much smaller set of lineages is that Fordham deliberately erred on the side of inclusiveness, taking a conservative approach to identifying gaps in apparent intergradation between phena, in this initial attempt at a lineage tree. Also, research subsequent to Fordham’s study of the late 1970’s, typified by the Paleogene Atlases [5, 55, 60], has led to the recognition of much more subtle discrimination of evolutionary information, including surface microstructure, morphometric analyses, and paleoceanographic ecogroups detected by stable isotopes.

## Data, topologies, and taxa of the 2011 study’s trees

### The data behind the trees

Aze & others [7] presented their phylogenetic trees in fully digital form (data and graphs). Their spreadsheet (Appendix S5 in [7]) of stratigraphic ranges (as dates) and ancestors provided the data needed to draw the morphospecies and lineage trees, and in each of two topologies: fully bifurcating (worksheets aMb, aLb respectively) and budding/bifurcating (aM, aL). The terms budding and bifurcating (e.g., [70]) refer to two alternative outcomes for an ancestor after a speciation event: in budding, a divergent descendant splits off the ancestor and the ancestor persists after speciation (Fig 1D in [7]); in bifurcation, the ancestor ceases at speciation by splitting into two descendants (Fig 1C in [7]). As used by Aze & others (page 911 in [7]), a budding/bifurcating topology allows either outcome for any speciation event on the tree (Fig 3A in [7]), whereas a fully bifurcating topology imposes a bifurcation for every speciation event (Fig 3B in [7]).

**Fig 4:**
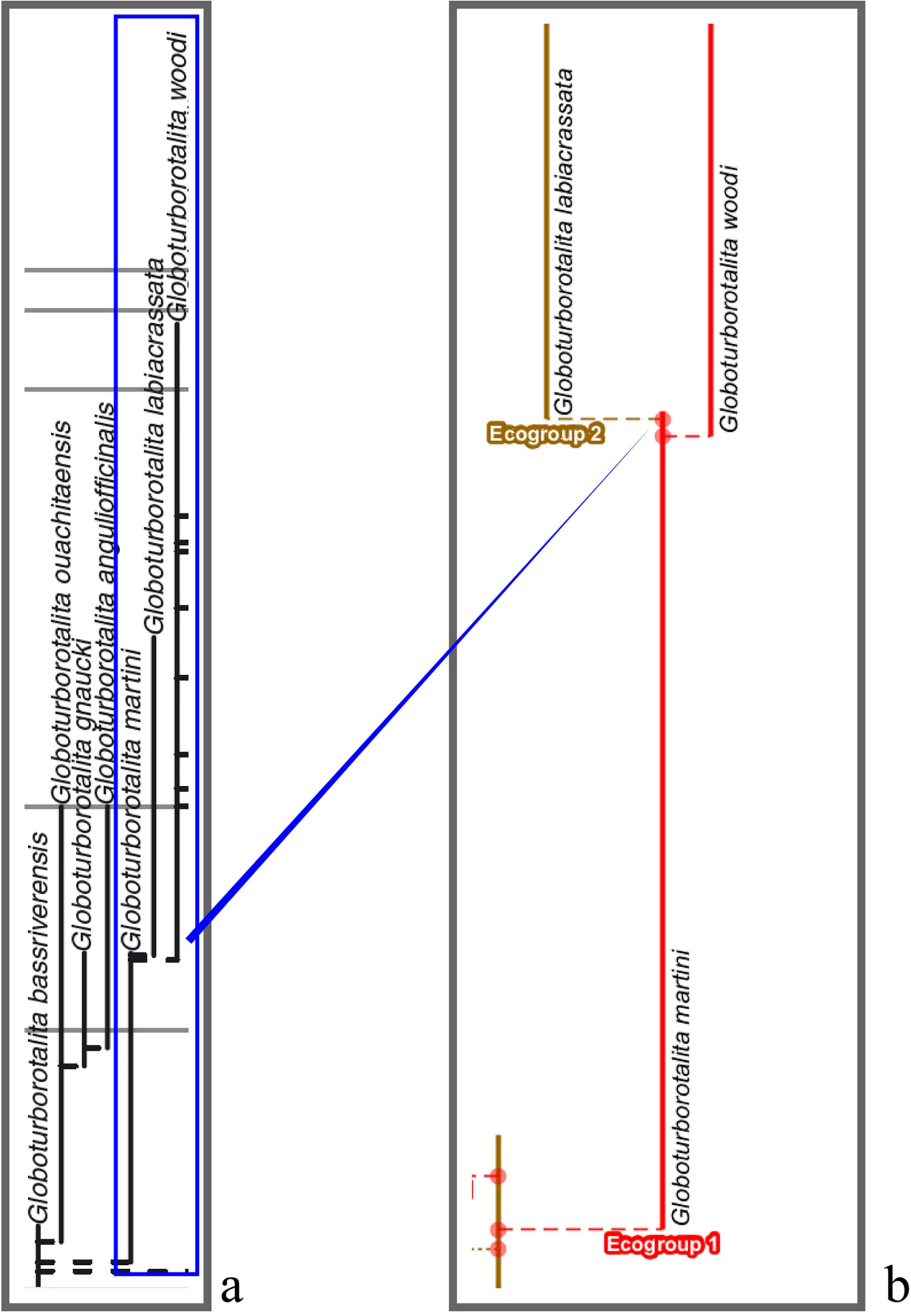
The budding/bifurcating morphospecies tree of the 2011 study—an example where the budding is close to a bifurcation. *Globoturborotalita woodi* and *G*. *labiacrassata*, both budding from ancestor *G*. *martini* near the uppermost limit of its stratigraphic range in the Rupelian (Oligocene). **a**, portion of the upper part of the morphospecies tree of Fig 5H in [7]. **b**, equivalent portion of transferred morphospecies tree [73], drawn using the evolutionary trees function of TimeScale Creator; red dots are branching points. The differences in the vertical scaling and positioning between **a** and **b** are merely for presentation. Similar examples from this tree include: *Subbotina cancellata* and *S*. *triangularis*, from *S*. *trivialis*; and *Morozovella praeangulata* and *Praemurica uncinata*, from *P*. *inconstans*.

**Fig 5:**
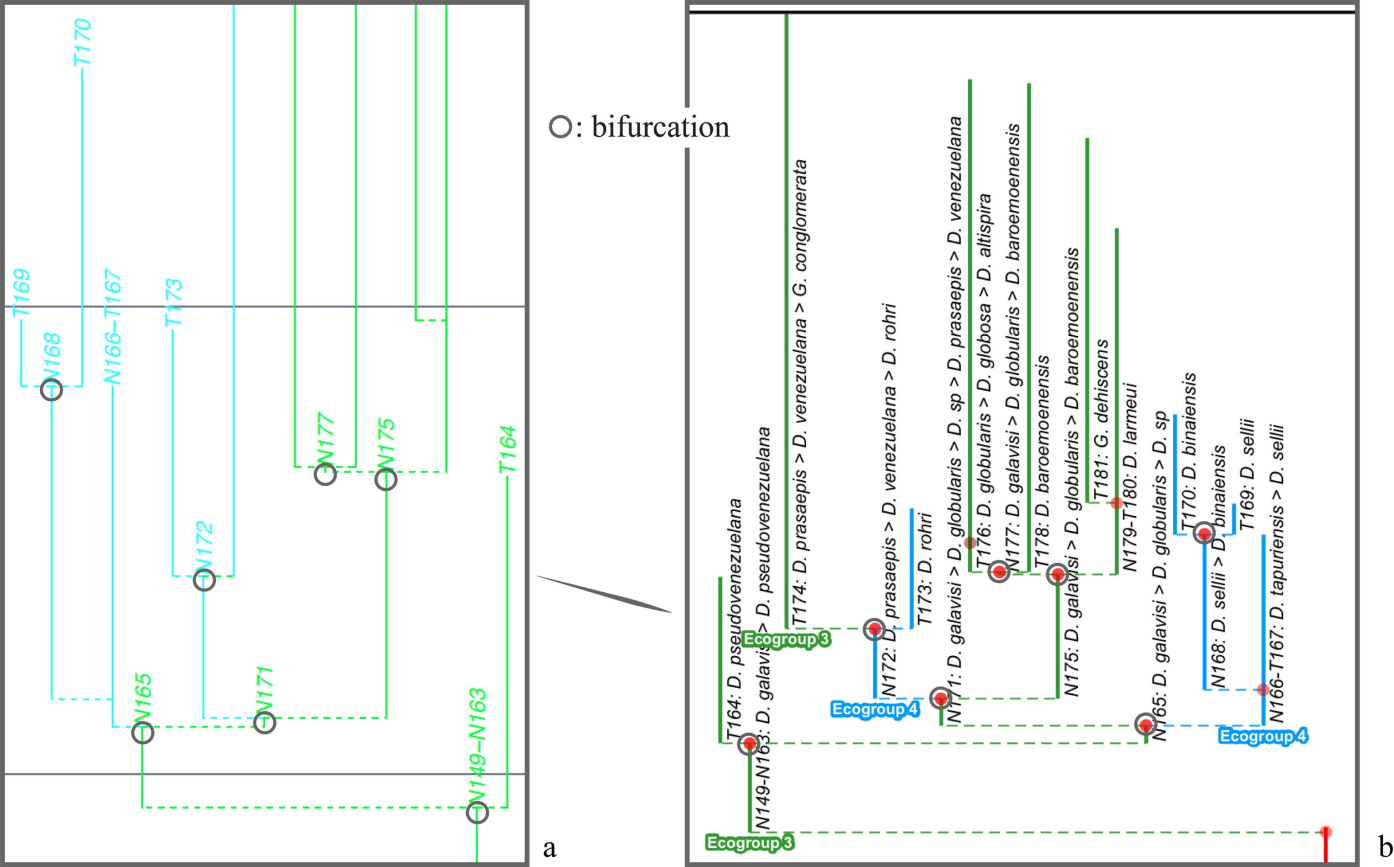
A part of the budding/bifurcating lineage tree of the 2011 study where a high proportion of bifurcations was interpreted. Early *Dentoglobigerina* (N149–N163) and descendant *Dentoglobigerina* and *Globoquadrina* lineages. **a**, portion of Appendix S2 in [7] (see Fig 3 herein); black lines, Eocene–Oligocene and Oligocene–Miocene boundaries. **b**, equivalent portion of the transferred lineage tree [74], drawn using the evolutionary trees function of TimeScale Creator (lineage codes augmented with morphospecies listing; see § Lineage labels); black line near top, the present. The differences in the vertical scaling and positioning between **a** and **b** are merely for presentation.

The trees were drawn (Figs 3–5 and Appendices S2 and S3 in [7]) as rectilinear evolutionary trees against geologic time (displaying Cenozoic epochs) employing the paleoPhylo package in R [71]. Associated information, including indications of morphology, ecology, geography, and literature sources, was provided as tables (Appendix S1 in [7]). The data, being in digital form, allowed calculation of divergence times of extant species (Appendix S4 in [7]) and quantitative comparison with the Neptune database to provide numerical assessments of the completeness of the fossil record of Cenozoic macroperforates (pages 917–919 in [7]). The 2011 study has since been employed as a key case example for quantitative macroevolutionary analyses (starting with [72]).

### Tree topologies

In this transfer to Timescale Creator only the budding/bifurcating topologies have been used for the morphospecies and lineage trees (from data in worksheets aM and aL of Appendix S5 in [7]). This was the preference of the 2011 work, in which the main body of the paper displayed only the budding/bifurcating trees in detail (Fig 5 in [7]). Although the fully bifurcating lineage tree was displayed in their Appendices 2 and 3, the fully bifurcating morphospecies tree was omitted there.

The budding/bifurcating morphospecies tree of the 2011 study did not actually contain any bifurcations; all were given as budding events, although some were very close to bifurcating (Fig 4). Most of the branching in the budding/bifurcating lineage tree was attributed to budding: of the 209 branching points (giving rise to the 210 lineages), only 50 were bifurcations (in pairs from 25 events), though they were common in some parts of the tree (Fig 5).

### Tree topologies and the study’s morphospecies and lineages

The selection of only the budding/bifurcating trees for this transfer to Timescale Creator, and the setting aside of the fully bifurcating trees, reflect peculiarities of taxonomy practised in the 2011 study which may not be clear to the broader biosystematic audience. For instance, given the preeminence of the notion of the lineage across diverse contemporary concepts of the species [54, 75, 76], the 2011 study’s retention of binomina only for morphospecies and the allocation of arbitrary codes to lineages might seem surprising. Also, the paper’s inclusion of fully bifurcating trees could imply that cladistic notions of common ancestry were involved in their stratophenetic phylogeny reconstruction, when they were not.

The 2011 study’s choice of morphospecies for formal species taxa was simply following conventional practice within the field of micropaleontology (page 903 in [7]). The field’s reliance upon rigorous typological taxonomy has long been considered a practical necessity especially in order to provide consistent marker taxa for biostratigraphic correlation, even though this has been guided by an evolutionary context from quite early in the field’s tradition (e.g., pages 38–44 in [28]; though the field, with few exceptions, continued to, and still does, stop short of acting on the implications of this context, see Chapter 4 in [77]). The dual morphospecies–lineage formal–informal taxonomy employed by the 2011 study can be likened to Mayden’s [54] demarcation between an ideal primary evolutionary species concept (the informal lineage of the 2011 study) and an operational secondary concept (the study’s formal morphospecies; Table 1 row 10).

**Fig 6:**
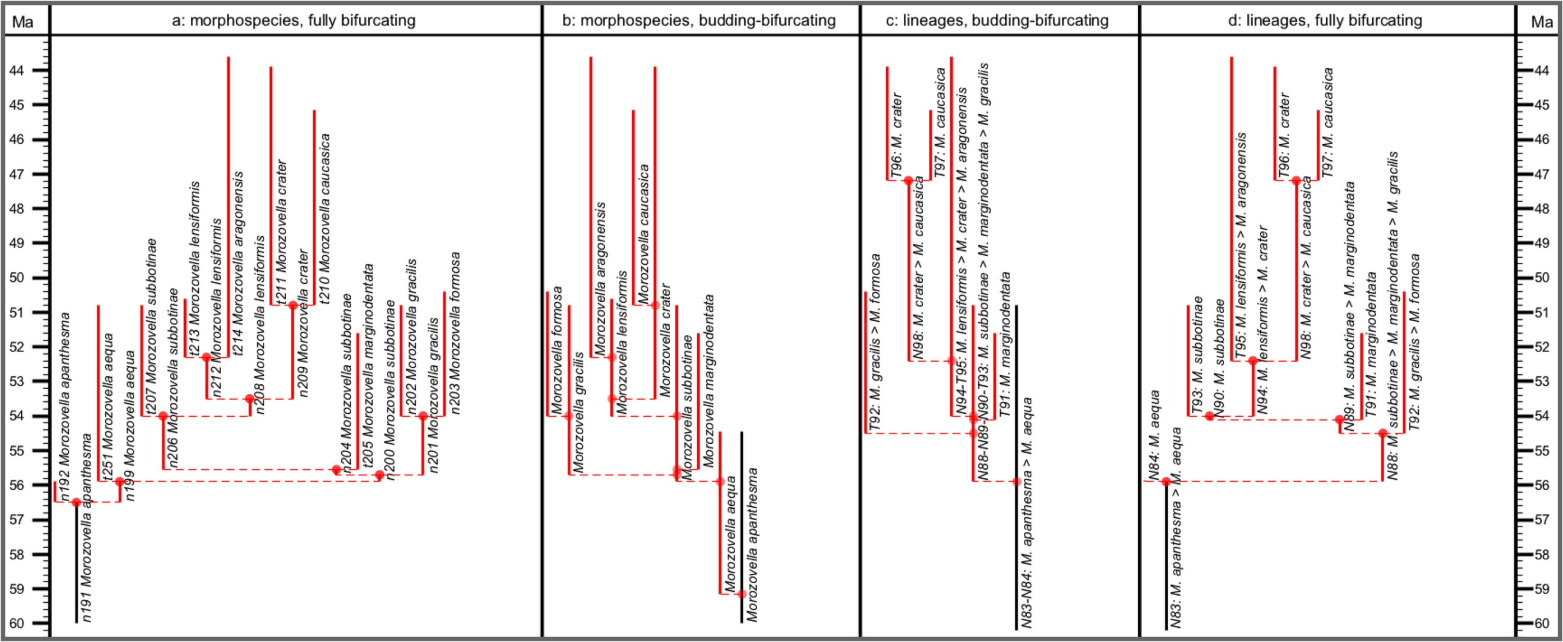
Comparison of the morphospecies and lineage trees of the 2011 study in each of the two topologies employed therein, budding/bifurcating and fully bifurcating. The example portion of the trees is after Fig 2 in [7], where the lineage tree (redrawn as **c** herein) was shown to be constructed from the morphospecies tree (redrawn as **b** herein), both displayed in budding/bifurcating topology. Here the corresponding fully bifurcating trees are added on either side: **a**, morphospecies tree; **d**, lineage tree. All trees drawn by the evolutionary trees function of TimeScale Creator using the data of Appendix 5 in [7]. (Unfortunately the trees from the 2011 paper and here differ, trivially, in some left–right placements of descendants above nodes, given the different spacings faced by the respective drawing programs.) Labels are the binomina and codes introduced in the 2011 study: **a**, morphospecies segment codes (n, internodal; t, terminal) for the fully bifurcating morphospecies tree; **b**, species binomina for the budding/bifurcating morphospecies tree; **c**, lineage codes for the budding/bifurcating lineage tree concatenated from the lineage segment codes of **d**; **d**, lineage segment codes (N, internodal; T, terminal) for the fully bifurcating lineage tree. Here all codes are followed by the binomina of the contained morphospecies for ease of comparison (see § Lineage labels). Note: we follow the contrivance of the 2011 paper to depict a bifurcating event in this example, by altering the budding of T97 to a bifurcation also including T96.

The fully bifurcating morphospecies and lineage trees of the 2011 study were not cladograms, but simply the result of restructuring their stratophenetic budding/bifurcating trees into internodal segments and labeling them with arbitrary codes (as a result the 2011 study employed only stratigraphic ranges from known occurrences, without consideration of range extensions, i.e., “ghost lineages” of [78] relevant to cladistic monophyly). Fig 6 provides an example from the 2011 paper where, for direct comparison, we present both the budding/bifurcating and fully bifurcating topologies.

These fully bifurcating trees can be considered artifactual—too much so, we feel, to be included in our transfer to Timescale Creator. This seems most sensibly argued with reference to lineages rather than morphospecies. As discussed in § Morphospecies tree, record-rich stratophenetics depends upon tracing usually stratigraphically very closely spaced ancestor–descendant links between specimen collections within lineages, below the extent and level of species taxa (that is, representing the populations-based species category as practiced in at least most taxonomic fields, not the morphospecies of conventional micropaleontology). The evidential focus on (“vertical“) stratigraphic linkages continues to apply not only along the extent of a lineage but also at putative branching events: for the latter, the stratophenetic evidence is assessed for the plausibility of a line or lines of descent from the ancestral lineage to either or each descendant lineage (as mentioned in § Morphospecies tree, the evidence is usually gathered in a low-tech observational manner, though it is sometimes subjected, albeit rarely, to sophisticated morphometrics, e.g., [68]). So for branching points in the tree, the degree to which the ancestral lineage may persist (morphologically, ecologically, etc.) into one or both descendant lineages is an important influence on and outcome of the phylogeny reconstruction. Although the encapsulation of this aspect of the branching event as either budding or bifurcating is necessarily a simplification, a budding/bifurcating topology at least preserves that approximation. A fully bifurcating topology, on the other hand, displays all branching points as discontinuous equal splits of the ancestor, erasing this aspect. The fully bifurcating topology in this context seems unnecessary for stratophenetically reconstructed trees, especially of the record-rich kind.

**Fig 7:**
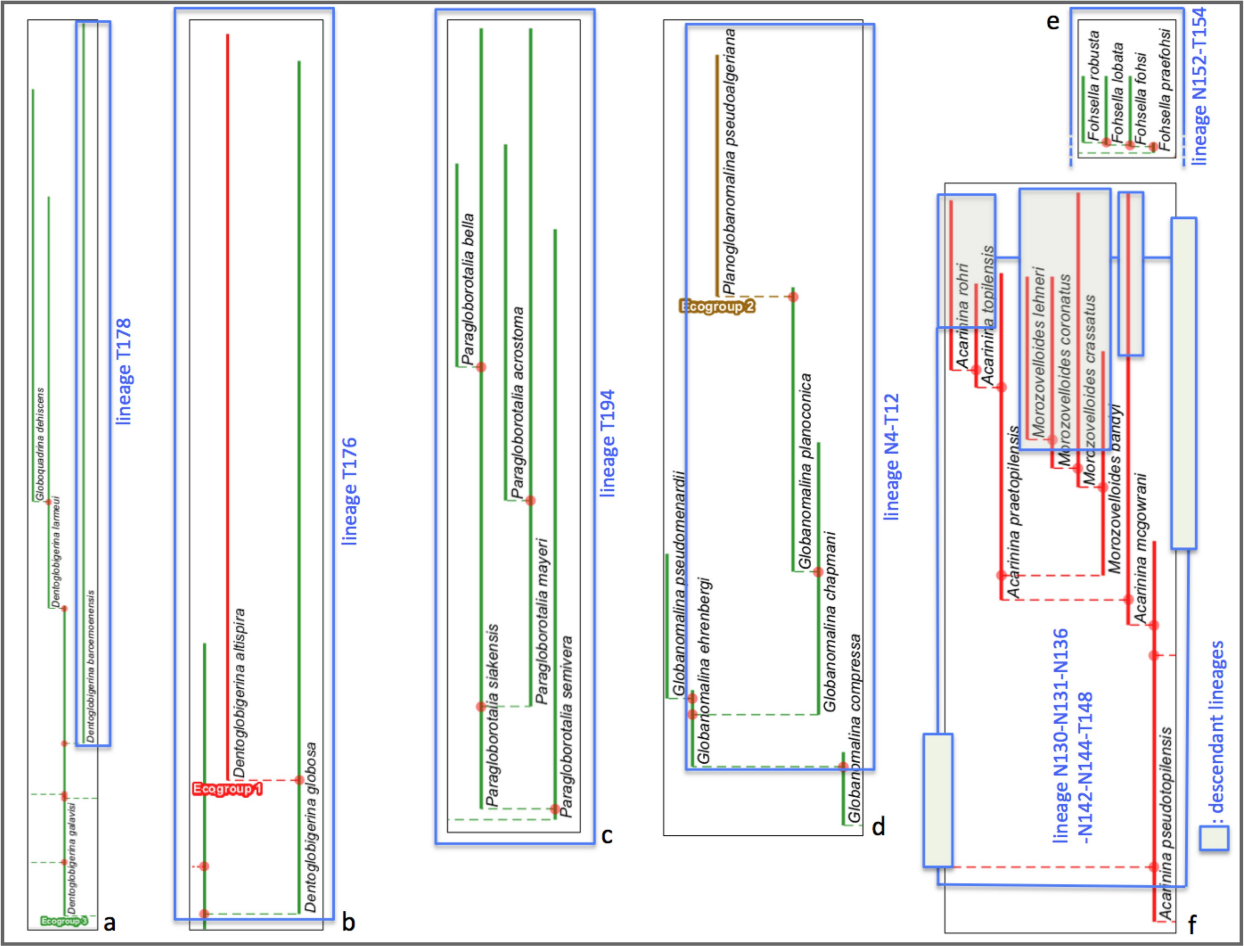
Parts of the morphospecies tree of the 2011 study exhibiting different evolutionary contexts for morphospecies. See Fig 2 in relation to Fig 3, herein; from the transferred morphospecies tree [82]. **a**, The morphospecies as a lineage: for most of its duration morphospecies *Dentoglobigerina baroemoenensis* constituted a long-lived almost unchanging monotypic lineage, T178, which persisted for ~23 Myr from the Oligocene to Pliocene. **b**, The morphospecies as a persistent but only one component of a long-lived almost unchanging polytypic lineage: for most of its ~23 Myr duration from the Oligocene to Pliocene, lineage T176 consisted of two morphospecies *Dentoglobigerina globosa* and *D*. *altispira*, favouring, respectively, open-ocean thermocline (green) and tropical and/or subtropical open-ocean mixed-layer (red) habitats; the persistence over such a long duration of these two morphospecies within the same lineage highlights the incomplete representation of the lineage by either of these morphospecies, within which either could, e.g., have partaken in an oceanographic cline and/or interbred with the other morphospecies. **c**, The morphospecies as a not-necessarily-typical component of a long-lived subtly changing polytypic lineage: throughout its ~20 Myr Oligocene–Miocene duration, starting with *Paragloborotalia semivera*, lineage T194 was polytypic but its morphologic content exhibited overall slow change which was not in any single direction and included intervals of high variability (e.g., five coeval morphospecies in the Burdigalian–Langhian of the Miocene); similar to example b, any one morphospecies is an incomplete representation of the lineage, but in this case more so given their only temporary membership and the nonsimple changing nature of the lineage. **d**, The morphospecies as only a snapshot of a polytypic lineage in which a similar morphologic trend was maintained over a long duration; over ~28 Myr in the Paleocene–Eocene, starting at the final occurrences of *Globanomalina compressa*, lineage N4-T12 was successively represented by five morphospecies with mostly little overlap in time; any one morphospecies represented only a short time interval for the lineage (approaching a paleospecies/chronospecies, see § Morphospecies tree) and a very partial snapshot of its properties. **e**, Similar to **d**, but the trend was rapid and short-lived: in the very last ~ 2 Myr of its ~ 10 Myr Miocene duration, lineage N152-T154 was progressively represented by four *Fohsella* morphospecies, three of which stayed and expanded its morphological variation; these morphospecies were very short-lived and highly partial snapshots of the lineage. **f**, The morphospecies was only one of several very different sources of morphological change and variation in the lineage, and also was shared with descendant lineages: in the Eocene, starting with *Acarinina pseudotopilensis*, lineage N130-N131-N136-N142-N144-T148 underwent slow to fast changes in morphology, accumulating variation or moving through morphological fields; the lineage gave rise to five morphologically quite different descendant lineages (not all morphospecies are shown here, including those of two descendant lineages, shown here without content), which share their starting morphospecies with this ancestral lineage [83].

The argument against the appropriateness of a fully bifurcating topology for the 2011 study’s stratophenetic lineage tree seems clearly stronger for the morphospecies tree. Evidence for continuity between morphospecies can be expected not only as ancestor–descendant links between stratigraphically successive specimen collections but also as morphological intergradation within specimen collections and across collections at a similar stratigraphic level. And this continuity may be maintained during and for some interval after the first occurrence of a descendant morphospecies constituting a morphospecies “branching” event (Fig 2B in [7]). A fully bifurcating topology for the morphospecies tree would interpret all such events as breakdowns in continuity not only between the two descendant morphospecies assigned to different morphospecies but also, even more implausibly, between ancestral and descendant parts of the same morphospecies (see Fig 6A). This is clearly a misrepresentation of the evidence gathered via record-rich stratophenetics. Rather, morphospecies and their connecting “branching” events were found in the 2011 study to exhibit a wide range of evolutionary patterns and not always related to lineages and their branching (Fig 7). These patterns ranged, for instance, from extended intervals of near-stasis, involving both monotypic (7a) and polytypic (7b) morphology, to examples of changing morphology through time (7c–f). The latter included changes both in the central morphology (replacement of morphospecies) and in the spread of variability (accumulation or loss of morphospecies), involving irregular (7c) to consistent trends that were slow (7d) or fast (7e). There were also many instances of morphospecies spanning and persisting for minor to considerable intervals from ancestral to descendant lineages (7f). These considerations go to explain why the 2011 study’s budding/bifurcating morphospecies tree lacked any exact bifurcations. It is also a reminder that the morphospecies of the 2011 study, despite their binomen labels, are of highly variable evolutionary value and are not suitable for macroevolutionary analysis that extends beyond their formulation as artificial morphologic segments of lineages (e.g., [79], in attempting to assess the relative roles of cladogenesis versus anagenesis in the fossil record, heroically applied the same and quite demanding model assumptions to all morphospecies of the 2011 study; see also [80, 81]).

## Transfering the 2011 study’s trees to TimeScale Creator: The “Corrected Version” and its database

The chief incentive for transferring the 2011 trees to TimeScale Creator is to preserve the currency of the 2011 dataset as a case study. A key element therefore is to prepare the stratigraphic ranges for recalibration, allowing the trees to persist authentically into the future as international time scales are updated. The transfer of course provides the opportunity to incorporate corrections or amendments as encountered during the transfer, and also to enhance the 2011 dataset in several ways such as more explicitly linking timings between the morphospecies and lineage trees. These objectives are accomplished mainly by developing a relational database for the dataset and presenting much of the information in it via the evolutionary trees function of TimeScale Creator. However, the underlying aim remains to preserve the contemporaneity and intent of the original study—it is a “Corrected Version”, not a revision.

A key advantage of a relational-database approach for the 2011 dataset is the clear separation of primary from derived information, especially useful in managing the interplay between morphospecies and lineages and in delineating and calibrating dates from different zonation time scales.

### Primary data and information: Database tables

**Fig 8:**
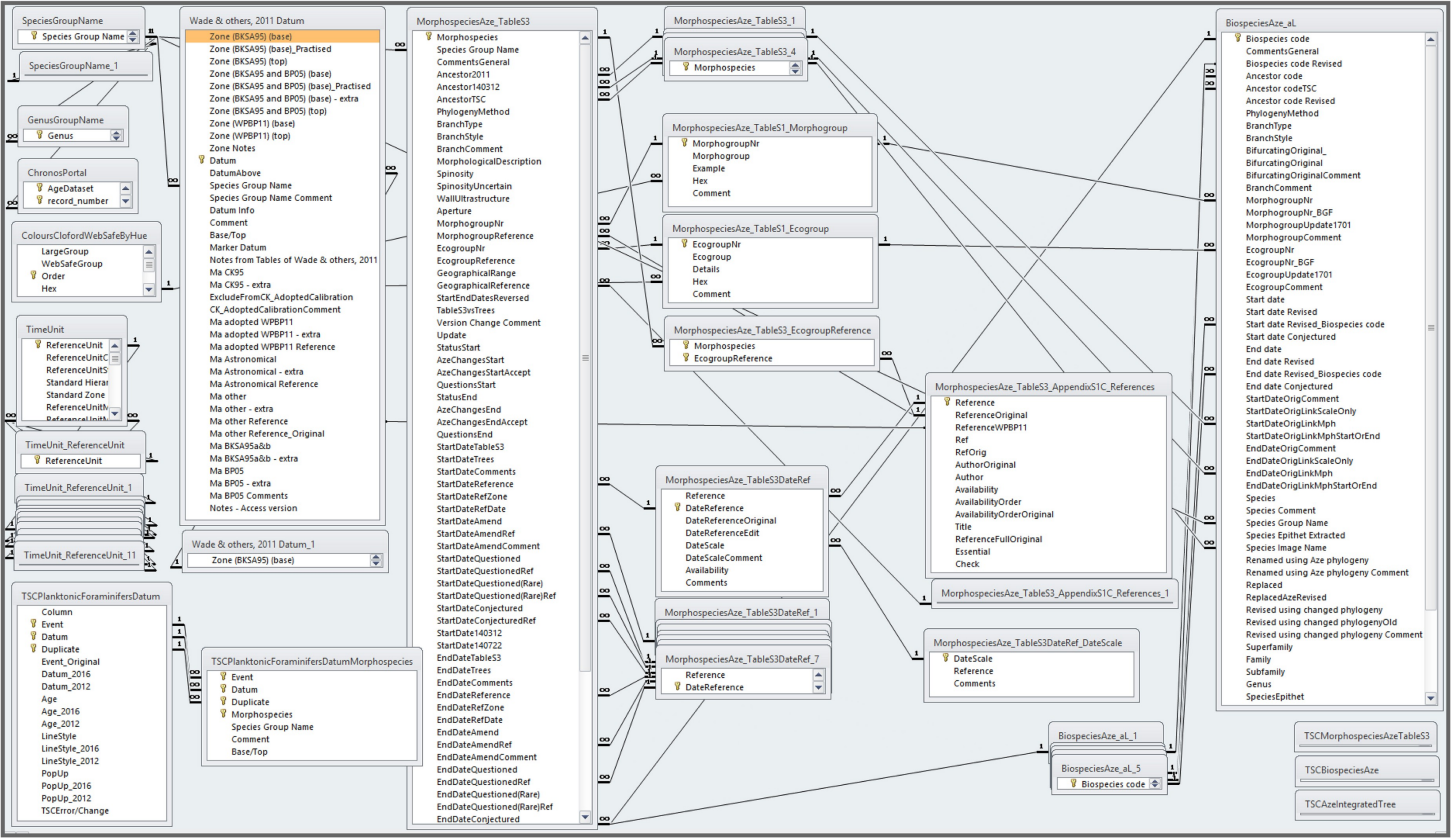
Tables and relationships in the database, TSCEvolTree_Aze&2011_CorrJul2018. 1-to-1 and 1-to-many (∞) relationships shown between key columns. This is a copy of the Relationships graphic within the database [84]; spreadsheet DBDesign in [85] provides extensive details of the design of the database.

The relational database, TSCEvolTree_Aze&2011_CorrJul2018, comprises two main tables, one for morphospecies, MorphospeciesAze_TableS3, the other for lineages, BiospeciesAze_aL (Fig 8 and Table 2 herein, [84], spreadsheets BasicData and DBDesign in [85]; see especially Table 2 herein for more details and examples to support the following discussion).

**Table 2:** Summary of relational tables in the TSCEvolTree_Aze&2011_CorrJul2018 database^b^.

**MorphospeciesAze_TableS3**
Details for the 339 morphospecies of the Aze & others paper [7], augmented from their Table S3 of Appendix S1 and worksheet aM of Appendix S5. The main focus is on clarifying the choice of stratigraphic ranges and ancestry, and incorporating post-publication corrections by the authors of Aze & others or selective corrections/amendments during conversion to TimeScale Creator.
Stratigraphic ranges are given as dates (in Ma); the time scales of the sources for the dates are made explicit (via links to table, MorphospeciesAze_TableS3DateRef). Almost all ranges are simple, as per those provided by the 2011 paper, delineated by lowest (start date) and highest occurrence (end date). However, a small number of ranges more closely represent those given by the nominated sources by also including range extensions: “questioned” or “questioned (rare)” for less confident stratigraphic occurrences; and “conjectured”, where a range extension is hypothesized, usually to support an ancestry proposal lacking contiguous stratigraphic occurrences. A proportion (~15%) of dates are corrected where minor differences in values were found between the 2011 paper and the nominated source; however, a systematic check was not conducted across the dataset. A further proportion (~15%) of ages are amended where alternative sources appear to better represent the intention of the 2011 paper; these include a few instances where there would be a conflict with the index (marker) datum sequence of the Wade & others [13] zonation. Corrections to dates are accompanied by brief explanatory comments. Minor changes to dates were also made by one of us (TA) for a proportion (~17%) of entries; most of these corresponded to the already invoked corrections or amendments.
Entries for ancestors follow the 2011 paper, with two exceptions in which adjustments to dates have removed the overlap in range between ancestor and descendant: a correction made by one of us (TA: for *Pulleniatina finalis*, *P*. *obliquiloculata* replaced *P*. *spectabilis*); and an amendment (for *Paragloborotalia pseudokugleri*, *Dentoglobigerina galavisi* is amended to *D*. *globularis*). Levels of evidential support for the ancestor–descendant proposals were not critically appraised as part of the TimeScale Creator conversion. However, column [PhylogenyMethod] was employed to distinguish a small number of proposals which were distinctly less (“not well”) or better (“strongly”) supported than the typical “well supported” proposals presumed for this group.
All other information given in Table S3 of Appendix S1 in [7] was incorporated, including indications of morphology, ecology, geography, and analyses made using the Neptune database. This information from Table S3 also included the lists of segments from both morphospecies (ID) and lineage (LID) trees within which each morphospecies occurred; in terms of relational logic, these could be supplanted by a single entry, the code for the lineage containing the highest occurrence of the morphospecies, and this was added manually for the TimeScale Creator conversion.
**BiospeciesAze_aL**
Details for the 210 lineages of the 2011 paper, augmented from worksheet aL of Appendix S5 in [7]. The main focus is to maximize and maintain consistency and transparency between morphospecies and lineages for dates of their stratigraphic ranges. This is achieved for the TimeScale Creator conversion by nominating a morphospecies whose date (start or end date) potentially defines the date (start or end) for a lineage; each morphospecies chosen for this is based on the apparent link between morphospecies and lineage dates used in the 2011 paper; this morphospecies is given by column [StartDateOrigLinkMph]. For start dates, ~40% of lineages could be linked in this way; for end dates, almost all (93%) could. Where a lineage range point of the 2011 study did not correspond to a morphospecies range point, then this morphospecies is at least used to provide the time scale applied to the date for the lineage.
Entries for ancestral lineages follow the 2011 paper, with two exceptions necessitated by changes in dates which place the ancestral lineage outside the time of origin of the descendant lineage: N150-N151-T153, involving the origin of morphospecies *Paragloborotalia pseudokugleri*; and N52-N54-T53, involving the origin of morphospecies *Hirsutella cibaoensis*. Levels of evidential support for the ancestor–descendant proposals were not critically appraised as part of the TimeScale Creator conversion. However, column [PhylogenyMethod] was employed to distinguish two proposals that were distinctly less (“not well”) or better (“strongly”) supported than the typical “well supported” proposals presumed for this group. The assignment of branching type as bifurcating or budding in the 2011 paper is incorporated.
Ecogroup and morphogroup allocations follow the 2011 paper (these data were not provided with the 2011 paper, but were indicated by colours employed in Appendices S2 and S3 in [7]; some colours for lineage morphogroups needed to be corrected; the ecogroup and morphogroup data for lineages were provided for the TimeScale Creator conversion by one of us [TA]). Some minor exceptions to these ecogroup and morphogroups were invoked for the TimeScale Creator conversion, in order to better match those of the contained morphospecies.
**MorphospeciesAze_TableS1_Morphogroup**
Details for morphogroups used for morphospecies and lineages; as for “Morphogroup” from Table S1 of Appendix S1 in [7], with explicit colour codes.
**MorphospeciesAze_TableS1_Ecogroup**
Details for ecogroups used for morphospecies and lineages; as for “Ecogroup” from Table S1 of Appendix S1 in [7], with explicit colour codes.
**MorphospeciesAze_TableS3_EcogroupReference**
Sources for ecogroups assigned to morphospecies; as for "Ecogroup reference", taken from Table S3 of Appendix S1 in [7]; multiple references in the original entries are accorded a row each.
**MorphospeciesAze_TableS3_AppendixS1C_References**
References for Table S3 of Appendix S1 in [7].
**MorphospeciesAze_TableS3DateRef**
Sources, and their time-scales, used for dates (sources from “Date reference” in Table S3 of Appendix S1 in [7]. The key purpose is to make explicit the time scale against which the source has (apparently) provided the date, essential in order to appropriately recalibrate to the current GTS time scale and also to maintain the capability to recalibrate to future time scales. An important example of this need is where dates from the Paleocene Atlas [5] have here been remeasured directly from the Atlas and so are against the time scale of Berggren & others [10], rather than calibrated to Wade & others [13] as in the 2011 study.
In the interests of transparency and to provide a pointer to recalibration steps needed, a further level of specificity is needed for those sources which imply more than one time scale for dates used. For the TimeScale Creator conversion, references to these sources also have the time scale specified. Examples include chapters from the Eocene Atlas [60]. For instance, in order for the TimeScale Creator conversion to record the questionable parts of the stratigraphic ranges given for some *Clavigerinella* morphospecies by Coxall & Pearson [86], additional start dates for these morphospecies have been measured directly from their Fig 8.1, drawn against the scale of Berggren & Pearson [23]. However, these dates need to be integrated with the dates from Coxall & Pearson already used in the 2011 paper, which were presented recalibrated by them to the scale of Wade & others. These two sets of sources are given as, respectively, “Coxall & Pearson (2006: BP05)” (against Berggren & Pearson) and “Coxall & Pearson (2006)” (against the time-scale option of Wade & others which was calibrated to Cande & Kent [9]). Analogous examples came from sources such as Berggren & others, which include some dates for which the usual recalibration is not applicable (reasons are specific to each instance and are indicated in comments fields in table, MorphospeciesAze_TableS3; spreadsheet DBDesign in [85] includes descriptions of these fields in worksheet, DesignMorphospeciesAze_TableS3, and corresponding data in worksheet, MorphospeciesAze_TableS3).
**MorphospeciesAze_TableS3DateRef_DateScale**
This simply gives full names for the four time scales requiring recalibration:
BKSA95: Berggren & others, 1995 [10]
BP05: Berggren & Pearson, 2005 [23]
WPBP11(CK95): Wade & others, 2011 [13]; calibrated to Cande & Kent, 1995 [9]
WPBP11(GTS04): Wade & others, 2011 [13]; calibrated to Gradstein & others, 2004 (GTS2004) [24].
**Wade & others, 2011 Datum**
Details for datums relative to zonations, compiled from Tables 1, 3, and 4 in [13].
Zonal (marker) datums are indicated, but other datums are also included, almost all of which provide intrazonal intervals employed for calibration between time scales. Datums specific to the BKSA95 zonation are separately tabulated from those of BP05, allowing calibration between zonations BKSA95, BP05, WPBP11(CK95), and WPBP11(GTS04) (see MorphospeciesAze_TableS3DateRef_DateScale, above). The WPBP11(GTS04) zonation corresponds to GTS2004 and so allows calibration to later GTS time scales (GTS2012, GTS2016).
Additional columns provide brief indications of adjustments needed for calibration, including a small number of alternative datums resulting from revised definitions of zonations. Nomenclatural links are provided for datum-naming taxa.
**Global tables:**
SpeciesGroupName
GenusGroupName
ChronosPortal
ColoursClofordWebSafeByHue
augmented from TimeScale Creator spreadsheet data:
TimeUnit_ReferenceUnit
TimeUnit
TSCPlanktonicForaminifersDatum
TSCPlanktonicForaminifersDatumMorphospecies
**Datapack tables:**
TSCMorphospeciesAzeTableS3
TSCBiospeciesAze
TSCAzeIntegratedTree

^b^(see also Fig 8 herein and spreadsheet DBDesign in [85])

The morphospecies table (Table 2; following Table S3 of Appendix S1 in [7]) compiles stratigraphic ranges and indications of morphology, ecology, and geography from stated sources, as well as stratigraphic analyses made using the Neptune database. The main focus of the morphospecies table for this transfer to TimeScale Creator is upon the ranges. This includes augmenting the table to record corrections or amendments to some dates (always within the provision not to revise but to better represent the intensions of the 2011 study) and, for a few ranges, to add questionable or conjectured range extensions. To enable the ranges to be recalibrated for subsequent time scales, we here link the date sources to an ancillary table (MorphospeciesAze_TableS3DateRef, Table 2) that explicitly indicates time scales for these sources. This measure also is needed to support the transfer because corrections or amendments made to dates from the 2011 study during the transfer are not necessarily against the time scale employed by that study (i.e., that of Wade & others [13], calibrated to Cande & Kent [9]). The measure also facilitates clear differentiation in the database where more than one time scale may, in effect, be applied by or to the source. Also added via the morphospecies table are indications of levels of evidential support for the ancestor–descendant proposals; these indications are generalized and not meant to suggest new information but rather to highlight a small number of demonstrably less or better supported proposals, and also to provide future capability for more deliberate attention to this aspect.

The main feature provided by the lineage table (Table 2) which augments the 2011 study is to embed in the table any links which may have been exercised manually in the 2011 study between the stratigraphic range of a lineage and that of an associated morphospecies; this applied to approximately 40% of start dates and almost all end dates of lineages. This is implemented in the database by assigning a morphospecies range point (start or end) to each lineage range point (start or end) and employing a field to turn on or off the link between the lineage and morphospecies range points; if this field is turned on, the lineage point adopts the date of the morphospecies range point; if turned off, the original date given in the 2011 study to the lineage point (or its replacement if corrected or amended) is retained but the time scale of the morphospecies point is employed for calibration of the date. This measure enables correspondences between the timing of the morphospecies and lineage trees to be made transparent but also to easily retain these linkages if morphospecies ranges are changed. A similar embedding feature has been added which allows the database to work out the lineage memberships of each morphospecies (see Table 2, table MorphospeciesAze_TableS3), thus obviating the need to manually construct these lists (and inevitably make human errors).

Another key table (Wade & others, 2011 Datum, Table 2) compiles the datums from Wade & others [13]. Here their tables are augmented to separately depict the zonations of Berggren & others (1995) [10] and Berggren & Pearson (2005) [23] in addition to the Wade & others zonation in its two versions (calibrated to Cande & Kent, 1995 [9], and to Gradstein & others, 2004 [24]). The remaining tables in the database include those (Global tables, Table 2) shared with other databases to provide, amongst other information, species and genus nomenclature, links to portals, and TimeScale Creator time units and datums.

### From the tables: Derived data and information, and datapacks

Much of the essential data needed for tree construction—names, dates, ancestors—are tabular or relational (or nearly so) and so mostly amounts to employing SQL queries to recast or combine elements from the database tables. This includes, from the main tables, the selection of ancestors, dates, and sources and associated commentary where, for instance, there are multiple options (e.g., corrected or amended entries), and then linking with accessory tables to add key determinants such as zonation time scales, accessory information such as taxonomic and grouping details, and paraphernalia such as colours.

Time-scale calibration of morphospecies and lineage dates employed a nested series of queries progressively recalibrating from those dates against the earlier published zonations to those against subsequent schemes, based on the augmented compilation of datums of Wade & others [13] (database table Wade & others, 2011 Datum, Table 2). Proportional calibration between the zonations of Berggren & others (1995) [10], Berggren & Pearson (2005) [23], and finally Wade & others, calibrated to Cande & Kent (1995) [9], employed zonal index (marker) datums only. So these calibrations, based only on sometimes relatively coarsely spaced planktonic-foraminiferal events, should be considered minimally adequate. For the jump to GTS2004, that is, from the scales of Cande & Kent (1995) to Gradstein & others (2004) [24], all datums of Wade & others were used, providing a finely tuned conversion, at least in terms of planktonic-foraminiferal events. Later GTS conversions, from GTS2004 to GTS2012 and finally GTS2016, employed planktonic-foraminiferal zonal index datums using tables augmented from TimeScale Creator spreadsheet data (Global tables, augmented from TimeScale Creator spreadsheet data, Table 2).

Programming, coded in the database’s Visual Basic, is then used to integrate tables and queries into derived tables from which TimeScale Creator datapacks [87] can be formulated. The programming includes procedural programming which is especially needed to generate the textural information provided in pop-ups as this is mostly nonrelational. Although the programming was developed inexpertly “in-house” and is code-intensive, it had the advantage of being able to be developed specifically to purpose and, being nonproprietary, able to be made available for scrutiny.

## Features of the Timescale Creator trees

**Fig 9:**
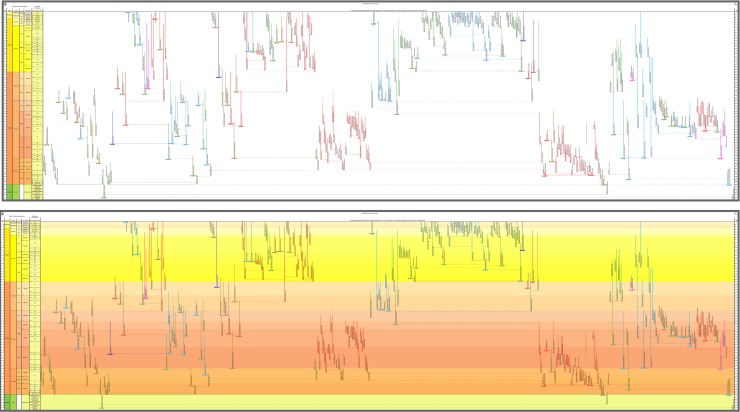
The transferred morphospecies tree of the 2011 study. “Corrected Version” (presented herein): budding/bifurcating topology; drawn using the evolutionary trees function of TimeScale Creator; ranges coloured and labeled by ecogroup. Overall view merely to convey the scale and temporal extent of ancestor–descendant groupings (corresponding to Fig 2). **Top**, white background (a full colour palette is available interactively in TimeScale Creator). **Bottom**, background coloured by Stages (“Chronostrat” setting: colours from the Commission for the Geological Map of the World).

**Fig 10:**
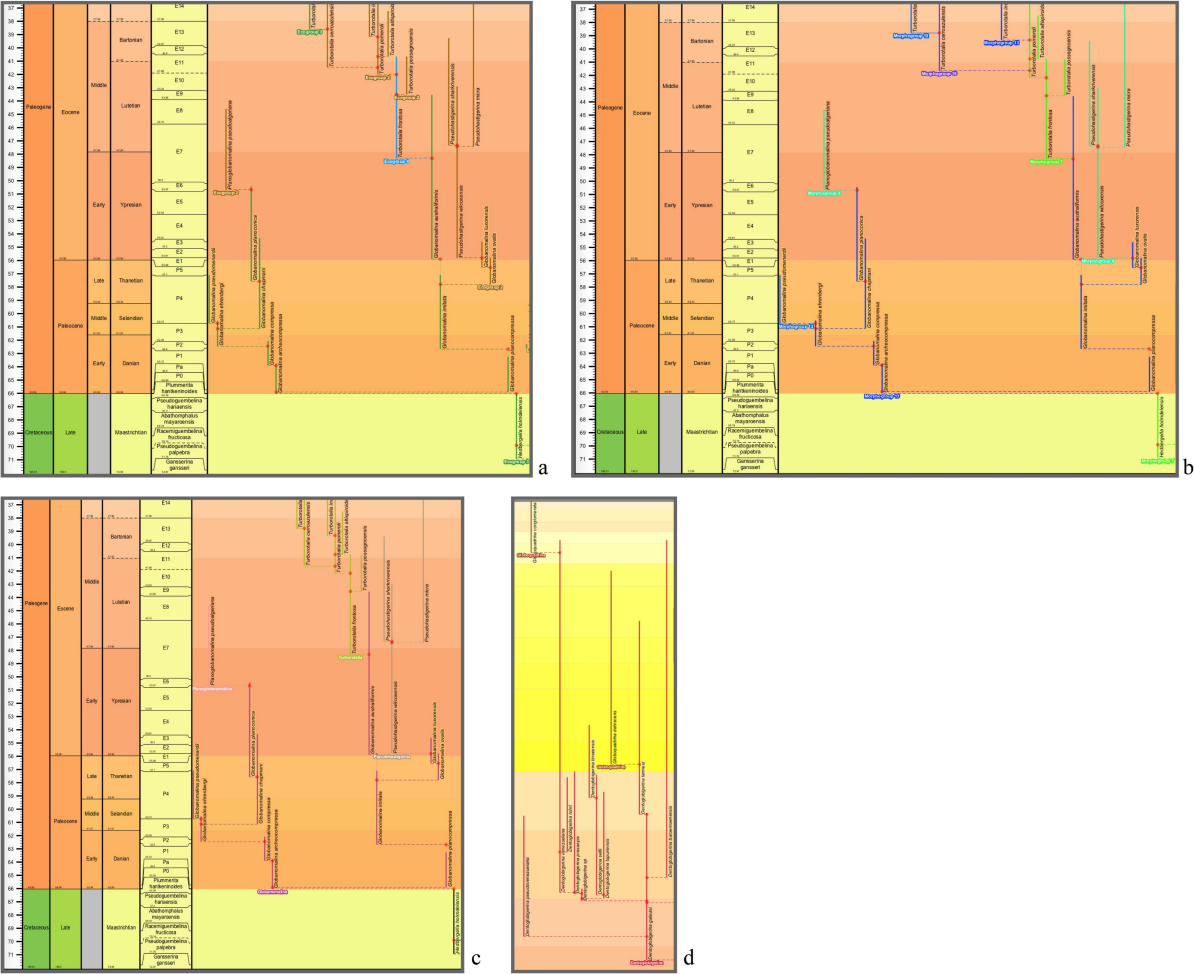
Close-ups of the transferred morphospecies tree. **a**–**c**, lower-left corner (Maastrichtian–Eocene) featuring *Globanomalina archeocompressa* and descendants. Ranges coloured and labeled by: **a**, ecogroup (as for Fig 9B); **b**, morphogroup; **c**, genus. **d**, a Bartonian (Eocene)–Holocene portion of the genus chart **c**, where the genus colours and labels accentuate the polyphyletic origin of *Globoquadrina* (as this genus was applied in the 2011 study). Note that, if desired, colours of range lines (and so their groupings) can be tracked visually more easily by various devices, e.g., zooming in, opting for a another background colour interactively, or selecting thicker range lines when programming or by editing the datapack.

The Timescale Creator platform is an interactive user environment in which the chart, in our case the evolutionary tree (see pages 22–25 and 88–92 in [88]), can be refreshed as options are selected (such as stratigraphic or Earth-history columns) and, given its vector graphics design, unlimited zoom and pop-ups can be employed on the fly. For example the morphospecies and lineage trees of the 2011 study can now be displayed against a time scale comprising not only standard epochs but a comprehensive gamut of Earth-history columns, not just the standard chrono- and bio-stratigraphic units figured here (e.g., left sides of Figs 9 and 10). And interactively, mouse-over pop-ups allow display of further details of these units (e.g., access to their up-to-date internationally agreed definitions).

### The trees overall

At the broadest view, the Timescale Creator versions of the 2011 trees can displayed against a monochrome background or projected against a background coloured by stages, the latter especially useful to keep track of the time interval while zooming in {see, respectively for the morphospecies and lineage trees, the white backgrounds of Figs 9 (top) and 11 (top), and their original 2011 equivalents, Figs 2 and 3; versus the stage backgrounds of Figs 9 (bottom) and 11 (bottom)}. A cursory look over the original 2011 and Timescale Creator versions of the whole trees (Figs 2 and 9 for the morphospecies tree; 3 and 11 for the lineage tree) reveals some variation in tree shape: for both sets of trees, spacing of clades is specific to each manifestation of a tree, it being determined by graphical programming algorithms dependent on, for example, the length of labels within the tree. A similar artifactual effect on tree shape to note (again, applicable to both sets of trees) is the left- or right-hand positioning (rotation) of descendant clades relative to ancestors (e.g., compare Fig 12A with 12B).

**Fig 11:**
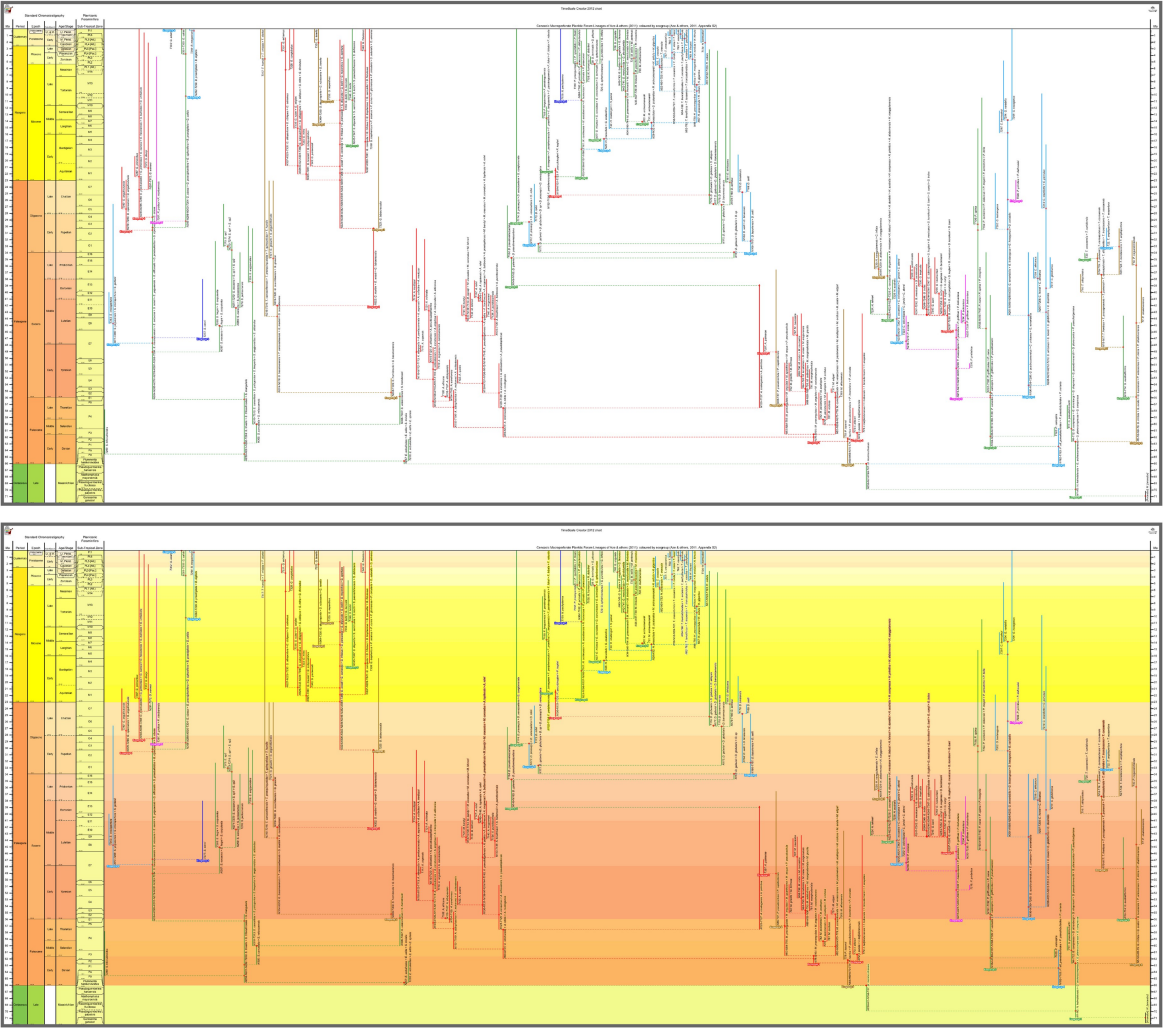
The transferred lineage tree of the 2011 study. “Corrected Version” (presented herein): budding/bifurcating topology; drawn using the evolutionary trees function of TimeScale Creator; ranges coloured and labeled by ecogroup. Overall view merely to convey the scale and temporal extent of ancestor–descendant groupings (corresponding to Fig 3 herein). **Top**, white background (a full colour palette is available interactively in TimeScale Creator). **Bottom**, background coloured by Stages (“Chronostrat” setting: colours from the Commission for the Geological Map of the World).

**Fig 12:**
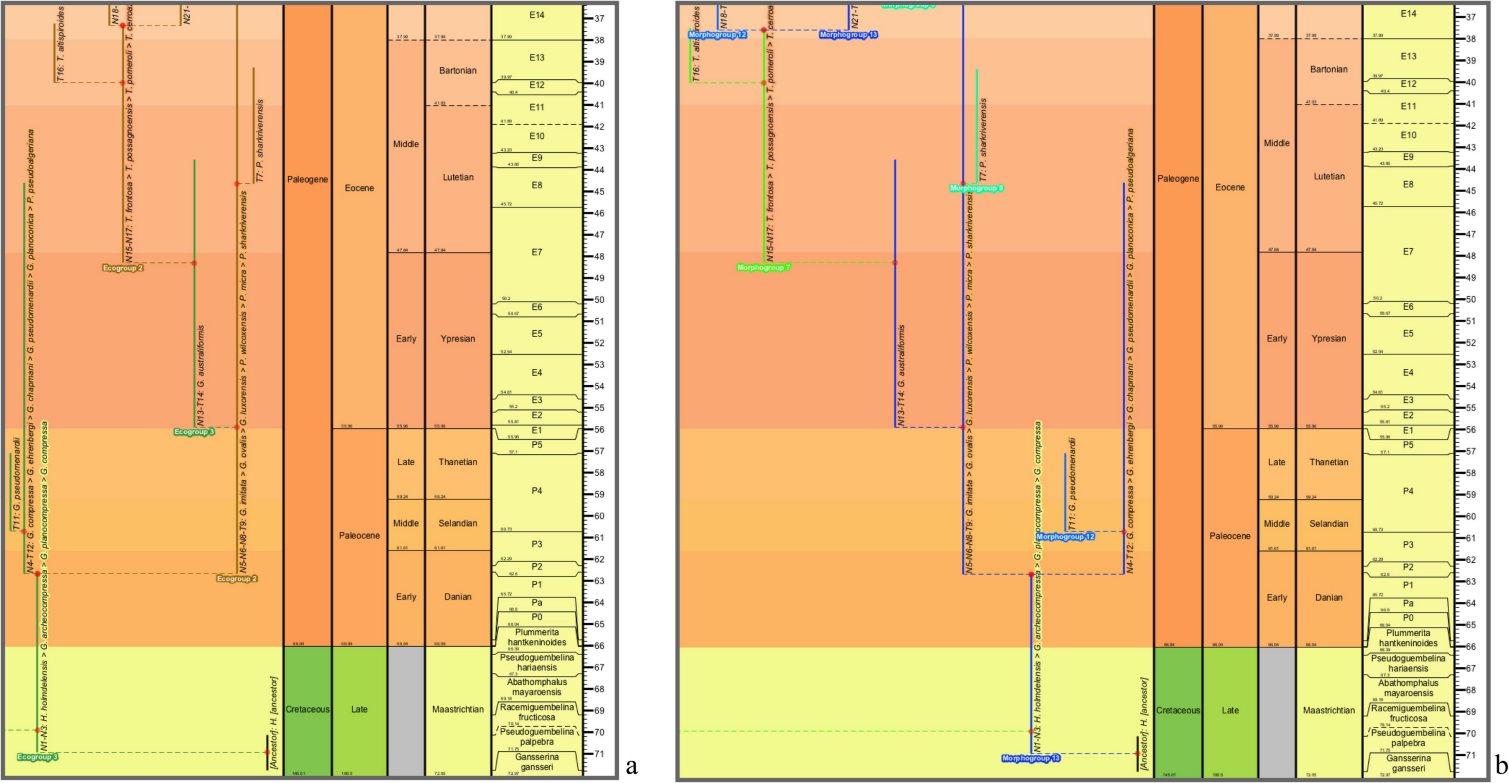
Close-ups of the transferred lineage tree. Lower-right corner (Maastrichtian–Eocene) featuring *Globanomalina* and descendants, starting with lineage N1-N3 (i.e., lineages corresponding to the morphospecies of Fig 10). Lineage codes augmented with morphospecies listing (see § Lineage labels). Range-line groupings coloured and labeled by: **a**, ecogroup (as for Fig 11A); **b**, morphogroup. Note that these portions of the ecogroup and morphogroup charts differ trivially in some left–right placements of descendants above nodes, given the different spacings faced by the drawing program. Also note the comment in the explanation of Fig 10 regarding tracking of colours of range lines.

### Timescale Creator–database customisation

Features provided by Timescale Creator enhance the information which can be gleaned from the 2011 trees. These features can be provided either from functions already built into Timescale Creator, or via “in-house” programming within the database which has exploited the built-in functions to provide data and information on key issues of interest to the case study. It is this flexibility provided by the combination of Timescale Creator functions and datapacks programmed from the back-end relational database which we hope to showcase now.

### Groups

Colours were used in the original 2011 trees (Appendices S2 and S3 in [7]), and now in the Timescale Creator trees, to display eco- and morpho-groups (respectively). The Timescale Creator trees also add coloured group labels (rather than colouring the range labels as in the original trees), and this allows identification of groups without recourse to the legend (see Figs 10A, 10B, 12A and 12B). These group labels are positioned on ancestor–descendant branches, but have here been programmed to display only when the group membership changes from ancestor to descendant. As a result, they have the added advantage of highlighting origins and reappearances of the selected groups or properties in a phylogenetic context. A handy use of this feature is when, for example, this is programmed to apply to the generic assignment of morphospecies (Fig 10C), making polyphyletic morphogenera, intentioned or otherwise, easy to spot (Fig 10D).

### Lineage labels

To label range lines on the lineage tree, the Timescale Creator version has been programmed to augment each lineage code with its list of contained morphospecies, e.g., the listing appended to Lineage N1-N3 is “*H*. *holmdelensis* > *G*. *archeocompressa* > G. *planocompressa* > *G*. *compressa*”(see Fig 12A). The morphospecies series in these listings is ordered by lowest occurrence, and so the >‘s denote stratigraphic succession. (The >‘s do not necessarily represent ancestor–descendant relationships; of course only a single line of descent could be expressed in such a format.) This allows the lineage and its proposed morphological succession to be grasped much more easily, including a ready comparison with the morphospecies tree (for Fig 12A, compare Fig 10A).

### Pop-ups

Pop-ups provide the most ample opportunity within Timescale Creator to provide access to supporting information for trees. Because pop-up windows are flexibly resizable and are coded in html, textual content has in effect few quota limitations and, in fact, can be employed to view external sources such as Internet sites and image files without the need to store them in the pop-up itself. They can also be programmed to follow a format tailored for the subject matter, as is done here.

**Fig 13:**
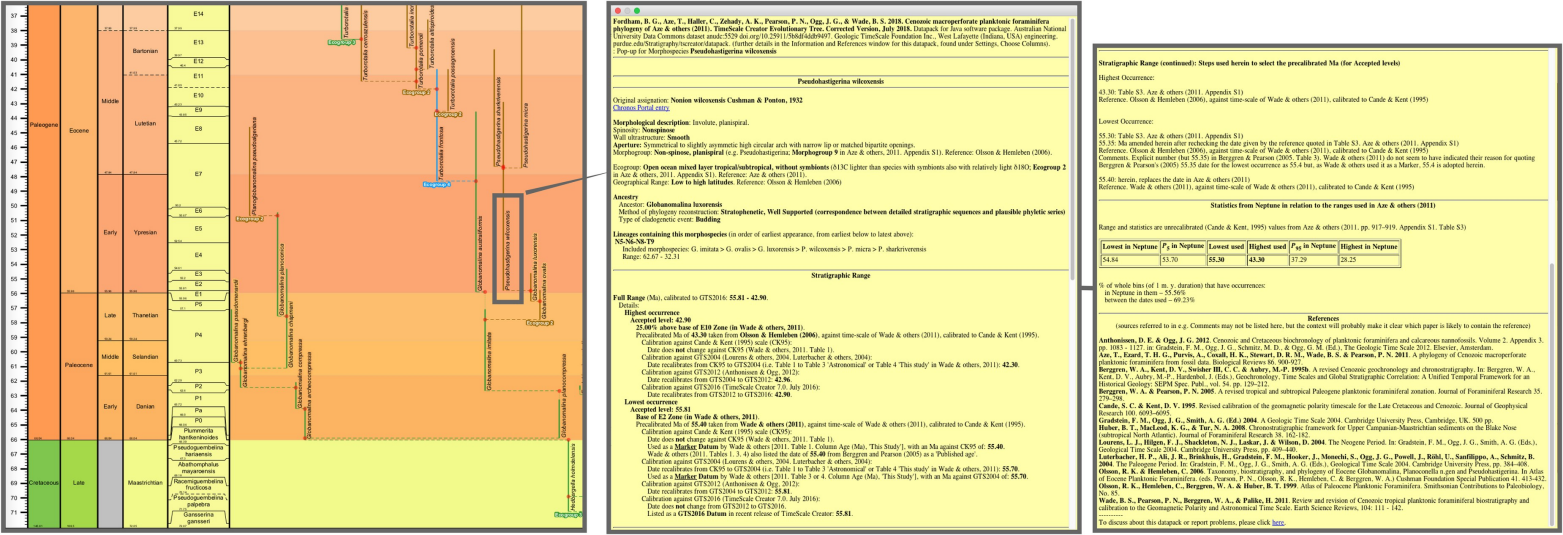
A pop-up from the morphospecies tree. **Left part**, close-up of the transferred morphospecies tree, as for Fig 10A; **middle and right parts**, pop-up (upper and lower portions of pop-up, respectively) for *Pseudohastigerina wilcoxensis*.

Pop-ups for the morphospecies tree (Fig 13) display the contents of the 2011 paper’s summary table (Table S3 of Appendix S1 in [7]), including decoding of eco- and morpho-group numbers, range statistics from the Neptune portal, and tailoring the reference list to each morphospecies. They also incorporate the ancestor (from worksheet aM of Appendix S5 in [7]), specify the type of cladogenetic event (all are, in fact, budding for this budding/bifurcating topology; see § Tree topologies), and level of support for the ancestor–descendant proposal (see § Branches). Lineages containing the morphospecies are listed, along with their morphospecies content and stratigraphic range (for details, see § Linkages between morphospecies and lineage trees [89]). Also included are the binomen’s original assignation and, where available, links to portals, the World Register of Marine Species (WoRMS) [90] and Chronos [91] (support for on-going activity on the foraminiferal section of Chronos no longer appears viable; other portals may need to be linked in later versions, e.g., pforams@mikrotax [92, 93]).

Details of stratigraphic ranges and their calibration occupy a major part of the morphospecies pop-up. This “Stratigraphic Range” section starts (Fig 13, middle) with the summary full range (in Ma) against GTS2016. Details are then given for the accepted lowest and highest points of the range (see § Range lines for ranges other than “accepted”): date; position relative to the standard planktonic-foraminiferal zonation; original date taken from its source and the source’s time scale; and then each of the steps of calibration leading to GTS2016, with an indication of any change in date at each step. If a calibration step for the range point was used as a datum in the zonation of Wade & others or in Time Scale Creator, this is indicated. The “Stratigraphic Range (continued)” section (Fig 13, right) appends brief details of multiple options which may have been considered to select the original date.

**Fig 14:**
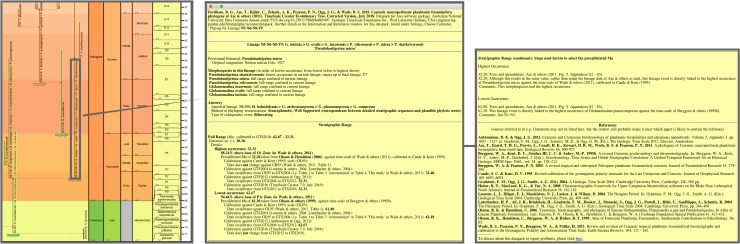
A pop-up from the lineage tree. **Left part**, close-up of the transferred lineage tree, as for Fig 12A (left); **middle and right parts**, pop-up (upper and lower portions of pop-up, respectively) for lineage N5-N6-N8-T9 (containing morphospecies *Globanomalina imitata*, *G*. *ovalis*, *G*. *luxorensis*, *Pseudohastigerina wilcoxensis*, *P*. *micra*, and *P*. *sharkriverensis*; i.e., the lineage containing the morphospecies, *P*. *wilcoxensis*, the pop-up of which is featured in Fig 13). Note that lineage N5-N6-N8-T9 extends higher, beyond the upper margin of this figure.

**Fig 15:**
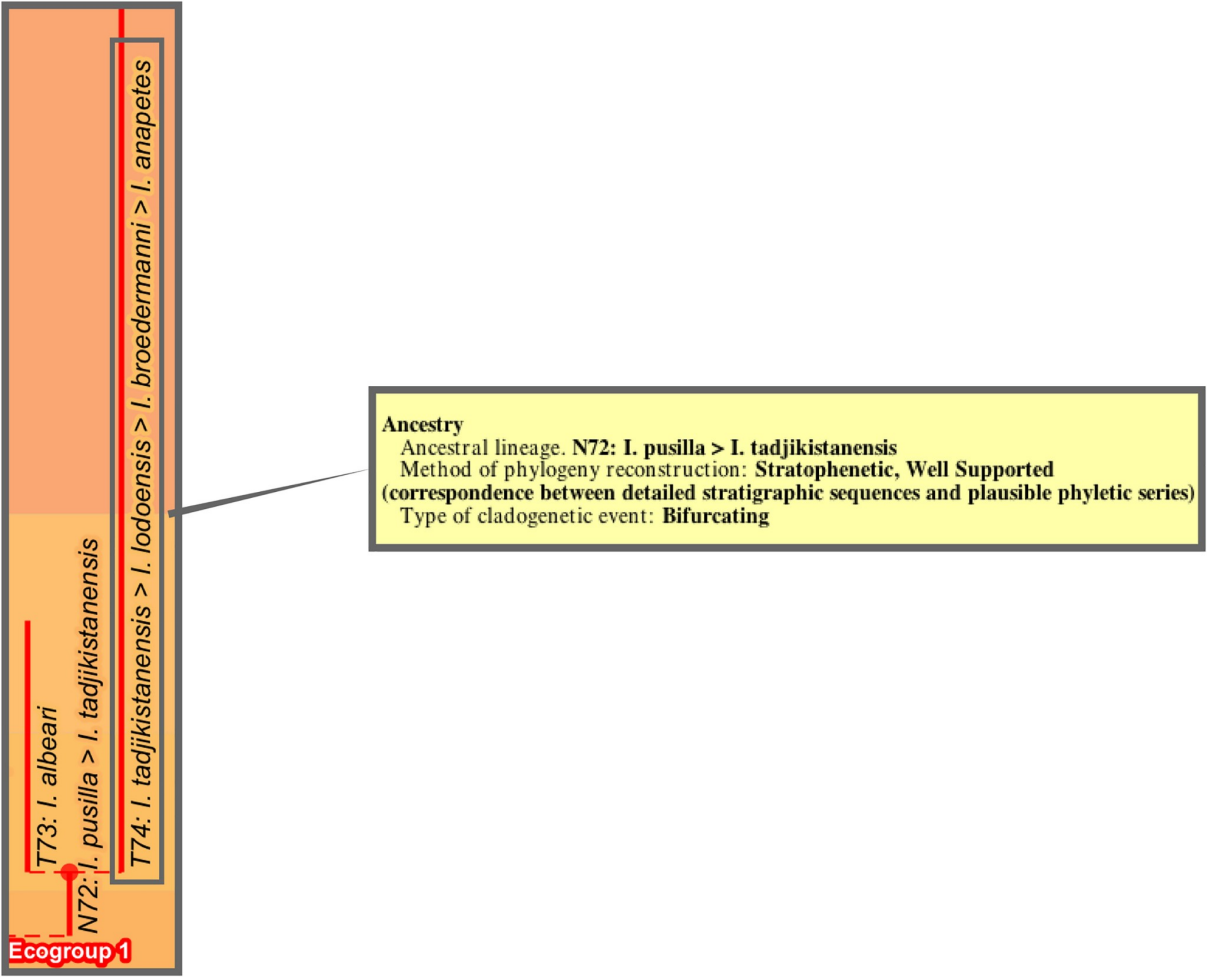
Bifurcating lineages. A minority of cladogenetic events for the lineage phylogeny was considered by the 2011 study to be bifurcating, rather than the prevalent budding. **Left part**: portion of the transferred lineage tree (Fig 11B herein); **right part**, “Ancestry” portion of the pop-up for Lineage T74. For the Selandian (Paleocene), Lineage N72 (containing *Igorina pusilla* and *I*. *tadjikistanensis*) was considered to split into Lineage T73 (containing *I*. *albeari*) and Lineage T74 (containing *I*. *tadjikistanensis*, *I*. *lodoensis*, *I*. *broedermanni*, and *I*. *anapetes*).

Pop-ups for the lineage tree (Fig 14) follow a similar format to the morphospecies, as appropriate [94]. For lineages, the 2011 study interpreted budding or bifurcating for the type of cladogenetic event for their budding/bifurcating topology (see § Tree topologies) and this entry is indicated under “Ancestry” (see Fig 15 for a bifurcating example). Morphospecies contained in the lineage are listed in order of lowest stratigraphic occurrence, and, for each morphospecies, the lineage from which it originates and the lineage in which it ends is indicated [95]. The “Stratigraphic Range (continued)” section of the pop-up, in the case of lineages (Fig 14, right), serves to detail the kind of link between a lineage range point and a morphospecies range point which has been embedded as part of the transfer to Time Scale Creator (for more details see § Linkages between morphospecies and lineage trees).

### Range lines

**Fig 16:**
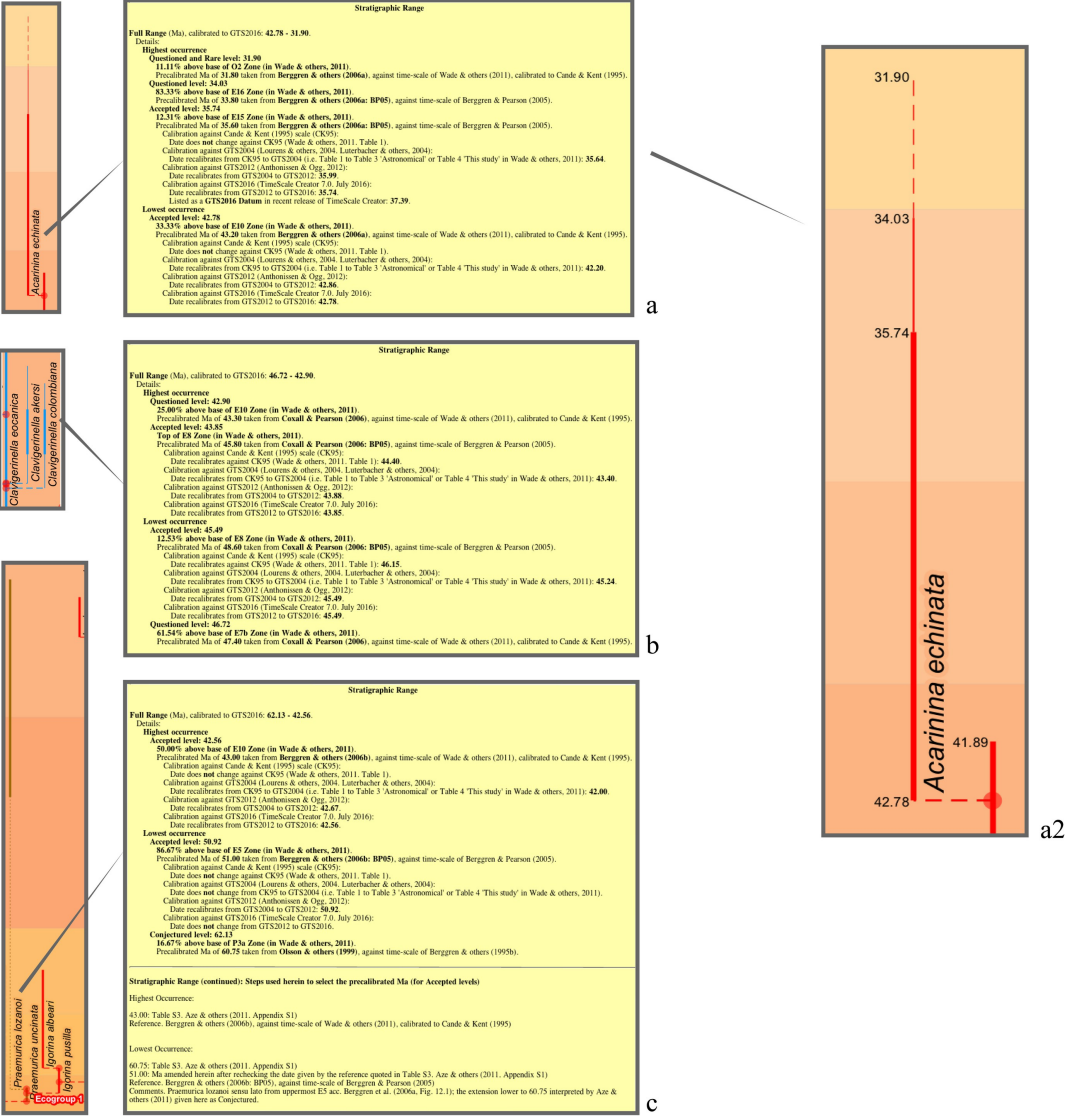
Range line styles and age labels, with supporting information in pop-ups. **a**–**c**, range line styles: levels of confidence for ranges—accepted, questioned, and questioned-and-rare stratigraphic occurrences, and conjectured extensions (not based on occurrences but hypothesized). **Left parts**, portions of the transferred morphospecies tree (Fig 9B); **right parts**, “Stratigraphic Range” portions of pop-ups. **a2**, range age labels: dates can be displayed along range lines (by checking option, Choose Columns, Show Age Labels). Age labels are given for all parts of a stratigraphic range—lowest and highest occurrences, and, if employed, accepted, questioned, questioned-and-rare, and conjectured levels. **a**, for the Lutetian–Rupelian (Eocene–Oligocene), the accepted range of *Acarinina echinata* (line of usual thickness) was extended [96] into later intervals based on questioned (thin line) and questioned-and-rare (broken line) occurrences. **b**, for the Lutetian (Eocene), the accepted ranges of *Clavigerinella akersi* and *C*. *colombiana* (lines of usual thickness) were extended [86] into both earlier and later intervals based on questioned occurrences (thin lines); with pop-up for *C*. *colombiana*. **c**, the accepted range of *Praemurica lozanoi*, based on occurrences in the Ypresian–Lutetian (Eocene) (line of usual thickness), was hypothesized [97] to extend into the Danian (Paleocene) but without evidence of occurrences (dotted line); with pop-up (including “continued” part) for *P*. *lozanoi*. **a2**, age labels for the range line of *Acarinina echinata*, from part **a**.

Range-line styles have been used for the Timescale Creator version of the 2011 trees to depict four levels of confidence for ranges. Apart from accepted ranges (lines of usual thickness), two less-confident records of stratigraphic occurrence are depicted: “questioned” (thin line) and “questioned-and-rare” (broken line; Fig 16A and 16B). For extensions to ranges that are not based on stratigraphic occurrences but are hypothesized (for various reasons), a “conjectured” range is separately recognised (dotted line; Fig 16C) to ensure that stratigraphic and hypothesized categories are not conflated. There is an option to attach age labels (in Ma) to range lines (Fig 16A2), providing the chart with an explicit deep-time positioning throughout.

### Branches

**Fig 17:**
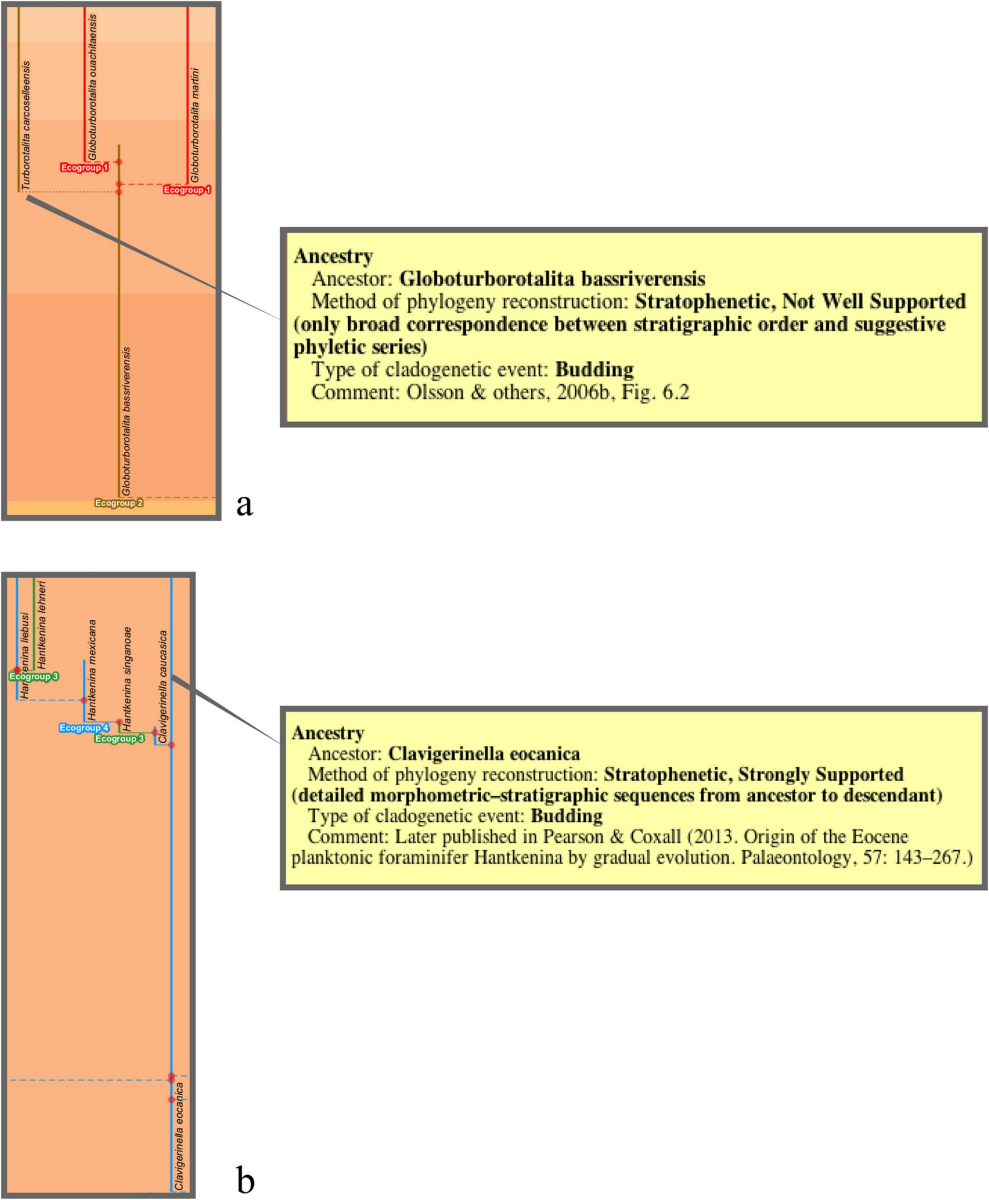
Branch line styles (morphospecies tree). Three categories of levels of support for ancestry. **Left parts**, portions of the transferred morphospecies tree (Fig 9B); **right parts**, “Ancestry” portions of pop-ups. **a**, “Not Well Supported” ancestry: for the Lutetian (Eocene), the descent of *Turborotalita carcoselleensis* from *Globoturborotalita bassriverensis* was questioned [64] (branch with dotted line); with pop-up for *T*. *carcoselleensis*; other branches “Well Supported” (branches with broken line). **b**, “Strongly Supported” ancestry: for the Lutetian (Eocene), a detailed stratophenetic and stable-isotope study [98] led to a sophisticated evolutionary model for the descent of *Hantkenina mexicana* from *H*. *singanoae*, in turn from *Clavigerinella caucasica*, and in turn from *C*. *eocanica* (branches with unbroken line); with pop-up for *C*. *caucasica*; other branches “Well Supported” (branches with broken line).

**Fig 18:**
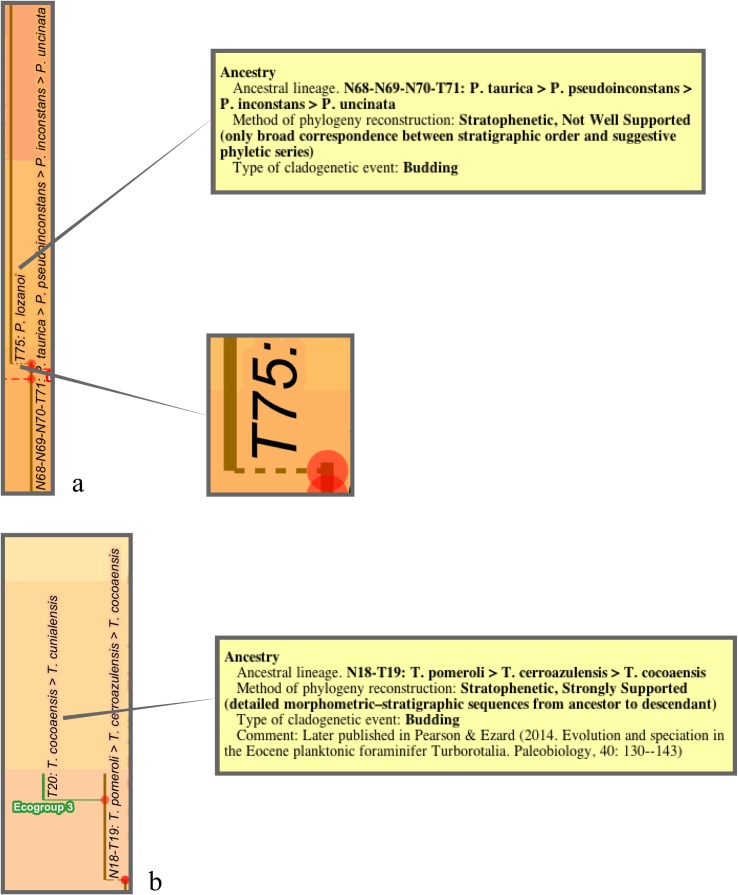
Branch line styles (lineage tree). Three categories of levels of support for ancestry. **Left parts**, portions of the transferred lineage (Fig 11B herein); **right parts (with yellow background)**, “Ancestry” portions of pop-ups. **a**, “Not Well Supported” ancestry: for the Danian (Paleocene), the descent of Lineage T75 (containing *Praemurica lozanoi*) from Lineage N68-N69-N70-T71 (containing *P*. *taurica*, *P*. *pseudoinconstans*, *P*. *inconstans*, and *P*. *uncinata*) was considered speculative (pages 397–398 in [97]; branch with dotted line; zoom-in shown); with pop-up for Lineage T75; other branches “Well Supported” (branches with broken line); see Fig 16C for a corresponding (though broader) portion of the transferred morphospecies tree. **b,** “Strongly Supported” ancestry: for the Priabonian (Eocene), detailed morphometrics [68] supported descent of Lineage T20 (containing *Turborotalia cocoaensis* and *T*. *cunialensis*) from Lineage N18-T19 (containing *T*. *pomeroli*, *T*. *cerroazulensis*, and *T*. *cocoaensis*; branch with unbroken line); with pop-up for Lineage T20; other branches “Well Supported” (branches with broken line).

Similarly to ranges, branch-line styles have been used to depict three levels of stratophenetic support for ancestry (Figs 17 and 18; see descriptions of database tables MorphospeciesAze_TableS3 and BiospeciesAze_aL in Table 2). Almost all ancestor–descendant proposals for the 2011 study are presumed to be “Well Supported” (correspondence between detailed stratigraphic sequences and plausible phyletic series; drawn as a broken line). A small number have been categorised as less or better supported than the usual: “Not Well Supported” (only broad correspondence between stratigraphic order and suggestive phyletic series; drawn as a dotted line); or “Strongly Supported” (detailed morphometric–stratigraphic sequences from ancestor to descendant; continuous line).

### Linkages between morphospecies and lineage trees

**Fig 19:**
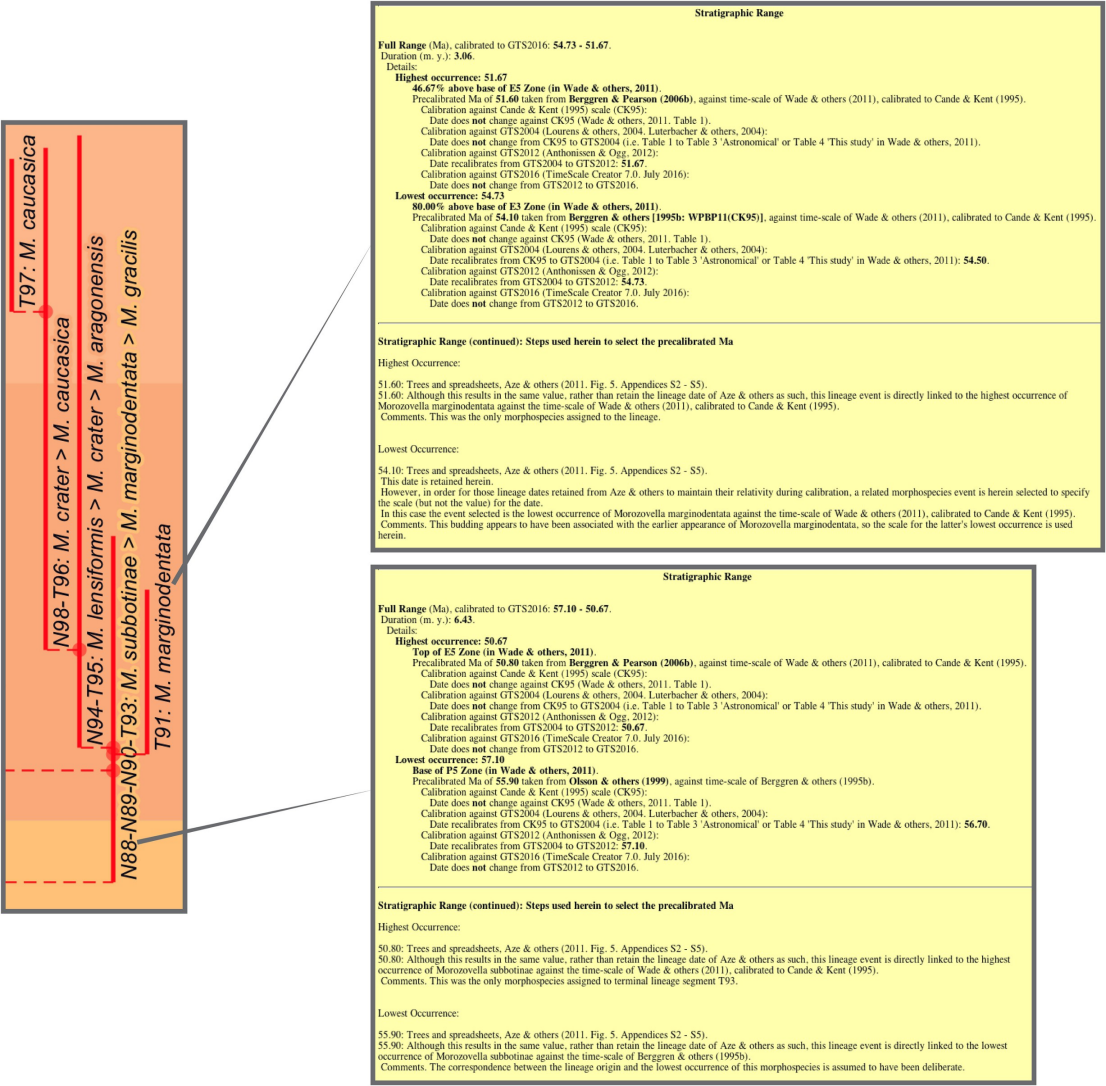
Using pop-ups to detail linkages between morphospecies and lineage trees, 1. Linkages invoked (or not) between a range point of a lineage and that of a morphospecies. Two Ypresian (Eocene) lineages (from Fig 2 in [7]; see also Fig 6 herein) exemplify budding of lineages linked, and not linked, to related morphospecies range points. **Left part**, portion of the transferred lineage tree (Fig 11B herein); **right parts,** “Stratigraphic Range” portions of pop-ups. **Lower right part**. Linking of lineages to morphospecies: the pop-up for lineage N88-N89-N90-T93. In the 2011 study the budding of lineage N88-N89-N90-T93 matched the lowest occurrence of *Morozovella subbotinae* (the origin of *subbotinae*–*marginodentata*–*gracilis*, Fig 2C in [7], lined up with that of *M*. *subbotinae*, Fig 2B in [7]; any morphological intergradation between *M*. *subbotinae* and *M*. *aequa* was of negligible duration on this temporal scale). The pop-up for lineage N88-N89-N90-T93 indicates that its Lowest Occurrence is herein matched to that of *M*. *subbotinae*, and now the (precalibrated) date for this lineage range point would follow any changes made to this morphospecies range point (for an instance of such a change, see the Lowest Occurrence of lineage N5-N6-N8-T9, Fig 14, right figure).**Upper right part**. Retaining a lineage range point, unlinked to that of a morphospecies: the pop-up for lineage T91. In the 2011 study the budding of lineage T91 from lineage N88-N89-N90-T93 corresponded to the breakdown of morphological intergradation between *M*. *subbotinae* and *M*. *marginodentata*, which was considered to have occurred later than the appearance of the latter (the origin of *marginodentata*, Fig 2C in [7], lined up with the upper limit of the grey intergradation interval drawn between *M*. *subbotinae* and *M*. *marginodentata*, Fig 2B in [7], but not with any of the morphospecies range points). The pop-up for lineage T91 indicates its Lowest Occurrence is herein retained and not directly linked to any morphospecies range point; nevertheless the retained date is employed against the time scale used for a nominated morphospecies point (in this case, the lowest occurrence of *M*. *marginodentata*).

Many range points of the lineages of the 2011 study are herein directly linked to those of included morphospecies: not quite half of start dates and almost all of end dates (see § Primary data and information: database tables). Brief details of this linkage are displayed in the “Stratigraphic Range (continued)” section of the pop-up, where the linkage will usually result in the same precalibrated date between lineage and morphospecies range points (see Fig 19, lower right), but these values will differ where there has been a correction or amendment of the original date (e.g., Lowest Occurrence, Fig 14, right). The reason for choosing the morphospecies range point is usually briefly indicated (e.g., Highest Occurrence, Fig 19, lower right: “Comments. This was the only morphospecies assigned to lineage T93”). Where the original date of the lineage range point is retained and not directly linked to a morphospecies point, the morphospecies and its time scale that are employed nonetheless for calibration are indicated (e.g., Lowest Occurrence, Fig 19, upper right).

**Fig 20:**
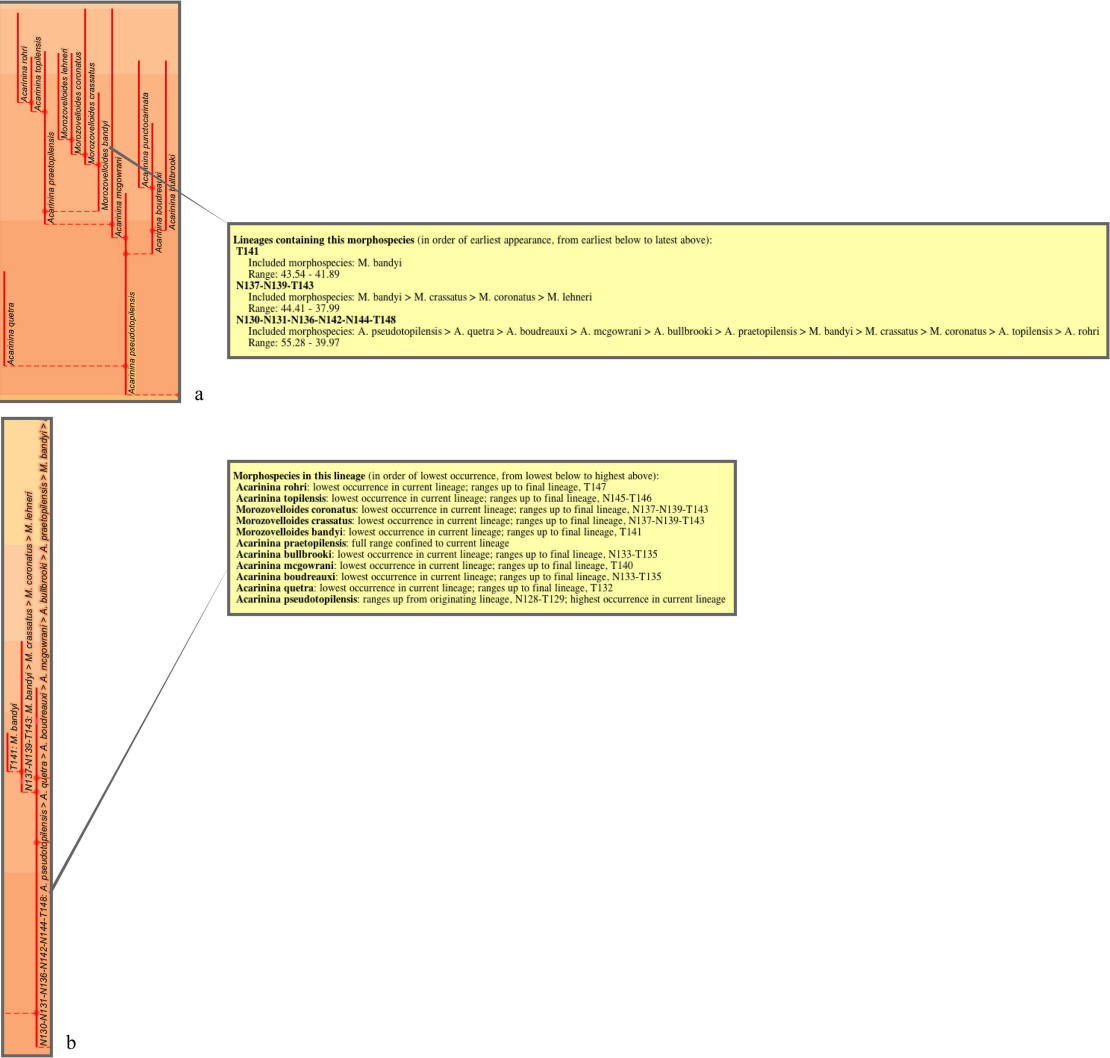
Using pop-ups to detail linkages between morphospecies and lineage trees, 2. Linkages following from the morphospecies content of lineages. This is exemplified by the morphospecies and lineages from Fig 7F, with special attention to the Lutetian (Eocene) *Morozovelloides bandyi*.**a**: **left part**, *Acarinina pseudotopilensis* and its descendants, a portion of the transferred morphospecies tree (Fig 9B); **right part**, pop-up of *M*. *bandyi*, the portion listing the three lineages in which *M*. *bandyi* occurs, and for each lineage its morphospecies content and range (in Ma).**b**: **left part**, the corresponding portion of the transferred lineage tree (Fig 11B) with the three lineages containing *M*. *bandyi*, N130-N131-N136-N142-N144-T148, N137-N139-T143, and T141; **right part**, the portion of the pop-up of the first of these lineages listing morphospecies in the lineage (including *M*. *bandyi*), ordered by lowest occurrence, each morphospecies with an indication of the lineage from which it originates and the lineage in which it ends.

Pop-ups are also employed to more easily appreciate the linkages between morphospecies and lineages, following from the morphospecies content of lineages. These are displayed both in terms of the lineages in which a morphospecies occurs (“Lineages containing this morphospecies”; see Figs 13 [middle] and 20A) and in terms of the morphospecies included in a lineage (“morphospecies in this lineage”; see Figs 14 [middle] and 20B), along with other information to help track these interrelationships (see explanation of Fig 20).

## Following on

The datapacks for the morphospecies and lineage trees of the 2011 study will be released on the TimeScale Creator website [17] (and on our data respository [87]) with the publication of this paper. Now that the trees have been transferred to TimeScale Creator, their ongoing currency, as a faithful record of the original 2011 study, will rely merely upon incorporating updates to the Geological Time Scale, a table update within the back-end database. The timing of these datapack updates would, as a minimum, follow major GTS releases and would similarly be made freely available on the TimeScale Creator website.

It is anticipated that a major revision of the trees will incorporate information from the recently published “Atlas of Oligocene planktonic foraminifera” [55] and is likely to warrant a publication summarising the changes and presenting datapacks for release on the TimeScale Creator website. With regard to other, especially future, research which improves or proposes alternatives to the trees, whether comprehensively or for subgroups, it is hoped that the trees already available, along with their databases and documentation, will encourage other teams to exploit and adapt these frameworks to their own ends and ideally share them via informal interchange, working groups, online file-hosting services, websites (including TimeScale Creator), and publications where warranted. Wider input from the research community will be a healthy test of especially the more-idiosyncratic and potentially ephemeral aspects of the 2011 study such as its lineage codes.

An additional feature for these trees, which would strongly aid their appreciation, would be to add images. If single images for each morphospecies or lineage were employed as thumbnails, attached to range lines, the trees would become a visual display of major morphological evolutionary trends and diversity (one of us, CH, compiled thumbnails for a draft 2013 version of the morphospecies tree [99]; it is hoped that this will eventually lead to an authoritative set that can be used in an illustrated datapack release). Additionally, pop-ups could be used to provide details for the imaged specimens; if a suite of images was provided for each morphospecies or lineage, the pop-ups could, for example, constitute the beginnings of digital systematic and/or stratigraphic atlases [67]. Of course, much more information could be incorporated into the database and displayed in pop-ups and/or output in other digital forms; discussion within the research community along these lines would be welcome.

The transfer of the 2011 trees into TimeScale Creator will also provide the opportunity to compare, in quite a precise context, this phylogeny side by side with alternative interpretations similarly transferred, as display of multiple datapacks is a routine feature. The alternatives could, for instance, be of historic interest, or of more-specific or broader systematic coverage, or from a different phylogenetic perspective such as molecular, or based on newer research as already alluded to above. It is hoped this will encourage a cross-pollination amongst a diversity of perspectives, whether that be scientific or technical.

## Conclusions

The study of Aze & others (2011) [7] has proved to be a key macroevolutionary dataset. Its time-scaled trees of Cenozoic macroperforate planktonic foraminifera, especially the first treatment of the lineages in decades, has led to well-fitting models of speciation and extinction rates related to diversity, species’ ecology, and climate change [72, 100]. These models, for both extant and fossil species, continue to inform a growing body of macroevolutionary literature ([100] has attained Web of Science’s Highly Cited Paper status). Our transfer of these trees onto the TimeScale Creator visualisation platform intends to maintain the currency of the 2011 dataset while, as much as has been practicable, preserving the original unrevised content of the trees: a “Corrected Version” which can serve as an historical but continually useable reference set.

The 2011 study presented two evolutionary trees for these macroperforates: a morphospecies tree and a lineage tree. The proposal of each of two taxonomic approaches to species will strike those from outside micropaleontology as unusual. The primary taxa were morphospecies binomina, largely traditional for the field but specifically representative of a relatively recent trend in micropaleontology towards consciously recognising species-level taxa as quite sizable segments of lineages. The study’s lineages were constructed by combining morphospecies along intervals in which they appeared to intergrade, and were labeled informally with codes concatenated from codes assigned to the included lineage segments. Both the morphospecies and lineage trees were transferred to TimeScale Creator.

The trees were not reconstructed from assessment of common ancestry of already defined taxa but from stratophenetic observations across very large collections of to-be-classified specimens, mostly from deep-sea oozes. This record-rich stratophenetics is the only available phylogenetic approach that can adequately exploit the rich stratigraphic record of planktonic foraminifera, though it can be usefully informed by ancillary approaches such as molecular analyses and cladistics. The 2011 study presented the trees in both fully bifurcating and budding/bifurcating topologies but only the latter topology was considered representative of the stratophenetic approach employed and so appropriate to be transferred.

Stratigraphic ranges of morphospecies and lineages were the key elements to maintaining currency of the trees against future geologic time scales. This was addressed by explicitly specifying time scales for the sources of the ranges of morphospecies, enabling transparent allocation of the original dates of the 2011 study to a zonation in the recalibration series. Consistency between the timing of morphospecies and lineage trees was improved by linking lineage range points to those of morphospecies where this was compatible with the 2011 study. These extra levels of rigour highlighted ranges or their sources needing corrections or amendments, including just a few instances where they were out of step with the standard zonation. The opportunity was also taken to refine the information on ranges or ancestor proposals. Two levels of questioned extensions to stratigraphic ranges were recognised to better represent a small number of reported instances, and a conjectured category was employed to distinguish a few examples where sources extended ranges beyond their known occurrences. Analogously, two levels of evidential support for ancestor–descendant proposals that were distinctly weaker or stronger than typical for these macroperforates were recognised for a small number of instances. These elaborations of details for ranges and ancestry are merely indicative attempts in this case but serve to encourage capture of more nuanced information from sources in the future.

Employing a relational database from which to generate the TimeScale Creator datapacks has allowed primary information to be clearly separated from derived information. Database tables house the primary information, mainly: a morphospecies table augmented from the 2011 study to provide the time-scaled ranges, ancestries, and ancillary information on morphology, ecology, and geography; a lineage table of ranges and ancestries, focused on providing links to the morphospecies ranges; and the datum tables of Wade & others [13], needed for calibration of pre-GTS2004 zonations. These primary tables constitute the core factual content of the trees and represent a resource in itself. Queries and programs serve to derive information from the primary tables, including calculating recalibrated dates, bringing together ancillary information from a number of core and global tables, especially useful for customising the largely nonrelational textual content for pop-ups, and compiling the datapacks.

The features displayed by the trees on the Timescale Creator platform are products of in-built functions as well as programming in the back-end database customised to exploit capabilities of TSC datapacks. Line styles allow depiction of questioned occurrences and conjectured range extensions and of ancestor–descendant branches proposed from atypically weaker or stronger evidence; not only can line colours display the extent of ancillary categories (eco-, morpho-groups) but also branch labels can highlight changes in categories, useful to, for example, warn of polyphyletic morphogenera. For lineages, range labels can be used to fill in the codes with their included morphospecies. However, it is the mouse-over pop-ups that provide the greatest opportunity to embed supporting information in the trees. Key are details for stratigraphic ranges and their recalibration steps, positions relative to the standard planktonic-foraminiferal zonation, and applications as datums. Other useful pop-up sections made possible by the relational structure of the back-end database include mutual listings between morphospecies and lineages, which ease the tracing of their interrelated contents, as well as details of any links recognised between the timing of lineage and morphospecies range points. Pop-ups also display and decode much of the ancillary information from the 2011 study’s summary Table S3, as well as basic nomenclatural references and links to portal entries.

All in all, the transfer onto the Timescale Creator platform of the morphospecies and lineage trees of Cenozoic macroperforate planktonic foraminfera of Aze & others (2011) should help ensure that this important dataset will now maintain its currency. It is also hoped that the development of the back-end relational database and the display of a range of supporting information on the trees will encourage greater understanding and critical scrutiny of the content of the trees and stimulate improved capture, presentation, and analysis of primary sources.
